# Exercise Therapy for People With Sarcopenic Obesity: Myokines and Adipokines as Effective Actors

**DOI:** 10.3389/fendo.2022.811751

**Published:** 2022-02-17

**Authors:** Hamed Alizadeh Pahlavani

**Affiliations:** Department of Physical Education, Farhangian University, Tehran, Iran

**Keywords:** sarcopenia, obesity, exercise, myokines, adipokines

## Abstract

Sarcopenic obesity is defined as a multifactorial disease in aging with decreased body muscle, decreased muscle strength, decreased independence, increased fat mass, due to decreased physical activity, changes in adipokines and myokines, and decreased satellite cells. People with sarcopenic obesity cause harmful changes in myokines and adipokines. These changes are due to a decrease interleukin-10 (IL-10), interleukin-15 (IL-15), insulin-like growth factor hormone (IGF-1), irisin, leukemia inhibitory factor (LIF), fibroblast growth factor-21 (FGF-21), adiponectin, and apelin. While factors such as myostatin, leptin, interleukin-6 (IL-6), interleukin-8 (IL-8), and resistin increase. The consequences of these changes are an increase in inflammatory factors, increased degradation of muscle proteins, increased fat mass, and decreased muscle tissue, which exacerbates sarcopenia obesity. In contrast, exercise, especially strength training, reverses this process, which includes increasing muscle protein synthesis, increasing myogenesis, increasing mitochondrial biogenesis, increasing brown fat, reducing white fat, reducing inflammatory factors, and reducing muscle atrophy. Since some people with chronic diseases are not able to do high-intensity strength training, exercises with blood flow restriction (BFR) are newly recommended. Numerous studies have shown that low-intensity BFR training produces the same increase in hypertrophy and muscle strength such as high-intensity strength training. Therefore, it seems that exercise interventions with BFR can be an effective way to prevent the exacerbation of sarcopenia obesity. However, due to limited studies on adipokines and exercises with BFR in people with sarcopenic obesity, more research is needed.

## Introduction

After the fifth decade of life, several changes in the body accelerate, including decreased mobility, increased muscle weakness, decreased muscle strength, increased adipose tissue, increased risk of falling and increased bone fragility, these factors are known as sarcopenia (1). In addition, some people with sarcopenia suffer from obesity called sarcopenic obesity because they are high in fat and low in muscle mass. There is evidence that sarcopenic obesity is associated with higher levels of metabolic diseases and increased mortality compared to obesity or sarcopenia alone (2). This phenomenon has much worse health consequences than people with only one disorder (3). More than 50 million people worldwide suffer from sarcopenia, and it is estimated that more than 200 million older people will be affected in the next 40 years. Annual skeletal muscle loss appears to be approximately 0.1-0.5% from age 30 but increases dramatically after age 65 (1). Sarcopenia is a multifactorial disease, which numerous studies have accepted many factors for sarcopenia, including decreased physical activity, reduced food intake, mitochondrial defects, increased chronic inflammation, increased proteolytic activity, decreased alpha nerve cells, hormonal disorders, decreased bone density, changes in adipokines and myokines, increased adipose tissue and decreased satellite cells (1). Among these factors may be decreased physical activity and reduced food intake as precursors of protein degradation that may exacerbate sarcopenia obesity.

Between the ages of 40 and 50, bone density slowly decreases by about 1-1.5 percent per year, muscle mass by about 1.5-2 percent per year, and muscle strength by about 2.5-3 percent per year (4). In addition, sarcopenia leads to mitochondrial DNA (mtDNA) mutations, increased release of reactive oxygen species (ROS), increased pro-inflammatory agents such as interleukin-6 (IL-6), and tumor necrosis factor-α (TNF-α), interleukin-1 (IL-1), as well as C-reactive protein (CRP) (1). Increased inflammation leads to the activation of protein degradation signals such as forkhead box O (FOXO), kappa B nuclear factor (NF-KB), and the ubiquitin-proteasome system in skeletal muscle, which is involved in the pathogenesis of sarcopenia (1, 5). These events appear to affect myokines that are effective in sarcopenic obesity such as IL-6, IL-10, IL-15, myostatin, insulin-like growth factor (IGF-1), irisin, and fibroblast growth factor-21 (FGF-21).

Decreased activity and increased caloric intake lead to adipose tissue hypertrophy, absorption of immune cells (macrophages), increase in cytokines such as TNF-α, IL-1β, and IL-6, and increase in inflammatory adipokines. These events cause muscle atrophy by increasing apoptosis (4, 6). Anatomically and physiologically, muscle and fat tissue are interconnected and play a key role in human metabolism. like the endocrine system, two organs communicate through myokines (derived from myocytes) and adipokines (derived from adipocytes) (4). Studies have reported direct adverse effects of adipokines on the myogenesis of human skeletal muscle myocytes, especially myocytes in the elderly. Anti-myogenic adipokines have inflammation effects that increase in obese people (1). Impaired regulation of adipokines is associated with sarcopenia. Increased adipose tissue mass in sarcopenic obesity is associated with major adipokines alterations such as leptin and adiponectin, and a high resistin/IGF-1 ratio (1). In addition, decreased muscle mass, increased fat mass, especially visceral fat, may lead to cardiovascular disease, increased insulin resistance, osteoporosis, atherosclerosis, nerve damage, and tumor growth (6).

Therapeutic interventions available for sarcopenic obesity include gene therapy, dietary supplements, physical activity, especially resistance training, anabolic hormones, and antioxidants (7). Exercise seems to be one of the best treatment options for sarcopenic obesity. For example, resistance training has been shown to affect body composition and physical function in patients with sarcopenic obesity and can be used to prevent muscle mass loss and physical problems in the elderly with sarcopenic obesity (8). Other studies on sarcopenia have reported that people aged 90 to 99 can increase their muscle strength by about 174%, their walking speed by about 48% with eight weeks of high-intensity progressive resistance training (9). In addition, after exercise intervention, sarcopenic obesity biomarkers such as weight loss and lean mass gain improved in the exercise group (10). A combination of resistance and aerobic exercise has also been reported to effectively reduce sarcopenic obesity and related biomarkers (8, 10, 11). Another study reported that after 24 sessions of resistance training with blood flow restriction (BFR), a 12% increase in muscle cross-section and an 8% increase in lateral extensor muscle thickness were observed in 90-year-olds. Finally, the researchers identified resistance training with BFR as an effective strategy for improving muscle mass and quality of life (12). In this study, we intend to investigate the effects of sarcopenic obesity and exercise on the mechanisms of myocytes and adipocytes. In the present study, we consider the main myokines and adipokines to find exercise mechanisms to combat sarcopenic obesity. Finally, this study is aimed to evaluate the effectiveness of exercise therapy for people with sarcopenic obesity.

## Myokines Affecting Sarcopenic Obesity

There is evidence that muscle-derived myokines play an important role in regulating muscle mass and function. Myokines abnormalities may underlie the pathogenesis of age-related diseases, including obesity, sarcopenia, and sarcopenic obesity (13). Myokines act as a mediator between skeletal muscle and other tissues (13). Myokines signaling pathways cause proliferation and differentiation of muscle cells, muscle atrophy, increased mitochondrial function, decreased inflammation, and metabolic homeostasis. These biological changes ultimately have a significant effect on muscle mass and physical function (13). Myokines affecting sarcopenic obesity include IL-6, IL-10, IL-15, myostatin, insulin-like growth factor (IGF-1), irisin, FGF-21, and leukemia inhibitory factor (LIF) (13).

## Interleukins

Interleukin-6 (IL-6) is secreted from both myocyte and adipocyte. IL-6, as a myokine, has a positive effect on skeletal muscle hypertrophy by regulating satellite cells (13). IL-6 also stimulates anti-inflammatory cytokines such as IL-10 and inhibits TNF-α pathway (6). In adipocytes of obese people, IL-6, as an adipokine, increase and is associated with obesity, inactivity, and inflammation (13). The constant increase in IL-6 level in adipose tissue regulates the activation of NF-KB transcription factors for protein degradation (6). In obese individuals, IL-6, as a pro-inflammatory factor, leads to decreased IGF-1 levels and decreased muscle volume and strength (6). TNFα, IL-1B, IL-1, and IL-6 also directly inhibit phosphoinositide 3-kinases (PI3Ks)/Protein kinase B (Akt) activity and protein synthesis pathway. In this way, IL-6 reduces skeletal muscle by signaling its receptor (14). In sarcopenia, there is an increase in the levels of inflammatory factors, including TNF-α, IL-1, and IL-6, and this increase is associated with a decrease in muscle mass. In particular, high levels of IL-6 and TNF-α are directly correlated with sarcopenia and weakness in humans and mice (15). Elevated IL-6 and CRP levels are also associated with loss of strength in aging (7). In confirmation of this, a study showed that increased plasma pro-inflammatory factors levels (TNF-α, IL-6, and IL-8) were associated with decreased strength in resistance training (13, 16). Under conditions of persistent inflammation and some diseases such as sarcopenic obesity, IL-6 is associated with muscle atrophy. In this way, depending on the location of secretion and physiological conditions, IL-6 acts as a double-edged sword (13). Thus, IL-6 is a significant marker of sarcopenia obesity in older (17). In response to exercise, the expression of IL-6 in the muscles increases (13). The IL-6 receptor also increases in muscle after exercise (18). After exercise, plasma IL-6 levels can also increase by up to 100-fold but are followed by increased expression of IL-10 and IL-1 receptor antagonists. This chronic response creates an anti-inflammatory environment in response to an increase in IL-6 circulation after exercise. IL-6 is involved in the oxidation of muscle triglycerides and blood sugar and stimulates lipolysis in adipose tissue during exercise (19). IL-6 has also been shown to have an inverse relationship with physical activity at the serum level (13). In the elderly and healthy subjects, progressive strength training reduces serum IL-6 levels compared with the control group (6). In general, after exercise, IL-6 as myokine leads to increased hypertrophy, increased lipolysis, and the formation of an anti-inflammatory environment, and a decrease in plasma IL-6 leads to a decrease in permanent inflammation.

IL-10 is released from T-helper cells, monocytes, and macrophages and regulates neutrophil activity. IL-10 released in muscle is an anti-inflammatory myokine (20). IL-10 plays an important role in altering muscle macrophages from phenotype M1 to M2 in damaged muscle, and this transfer is essential for normal muscle growth and regeneration. IL-10 prevents inflammation by suppressing macrophage activation as well as TNF-α, IL-2, IFN-γ, and IL-6. IL-10 improves age-related inflammation, obesity, and oxidative stress in skeletal muscle (13). In addition, mice lacking IL-10 show an increase in NF-KB, and IL-6 levels increase significantly after 50 weeks. IL-10-deficient mice reduce skeletal muscle strength with age and become attractive models for sarcopenia (21). There is evidence that levels of IL-6, IL-10, and the IL-6/IL-10 ratio increase in people with sarcopenia (13). Some studies have shown that IL-10 levels increase in older mice (22), and others have shown an increase in serum IL-10 levels in older humans (23). People with sarcopenia have higher levels of IL-6, IL-10, and visceral adipose tissue than people without sarcopenia (24). IL-10 in the serum is positively associated with obesity in humans (25). It seems that a compensatory increase in IL-10 in people with sarcopenia obesity is to counteract chronic inflammation (13). In contrast, combined exercise in people with sarcopenia increase thigh cross-sectional area and IL-10 and decreased TNF-α (20). Exercise in obese mice has additive effects on plasma IL-10 (26). Obese people decrease TNF-α and IL-6 after 12 weeks of exercise, while they increase adiponectin and IL-10 (27). Exercise reduces TNF-α protein (26%) and mRNA (58%) compared with the inactive group while increasing IL-10 expression (2.6-fold) and IL-10/TNF-α ratio in Mice. This study also reports that the long-term anti-inflammatory role of IL-10 is more pronounced under pathological conditions of low-grade inflammation (28). It is reported to exercise releases IL-10 from skeletal muscle into the bloodstream in people with sarcopenia obesity (25). Therefore, it is expected that by increasing IL-10 through the repetition of training sessions, a more anti-inflammatory environment for protein synthesis will be created.

**Figure 1:**
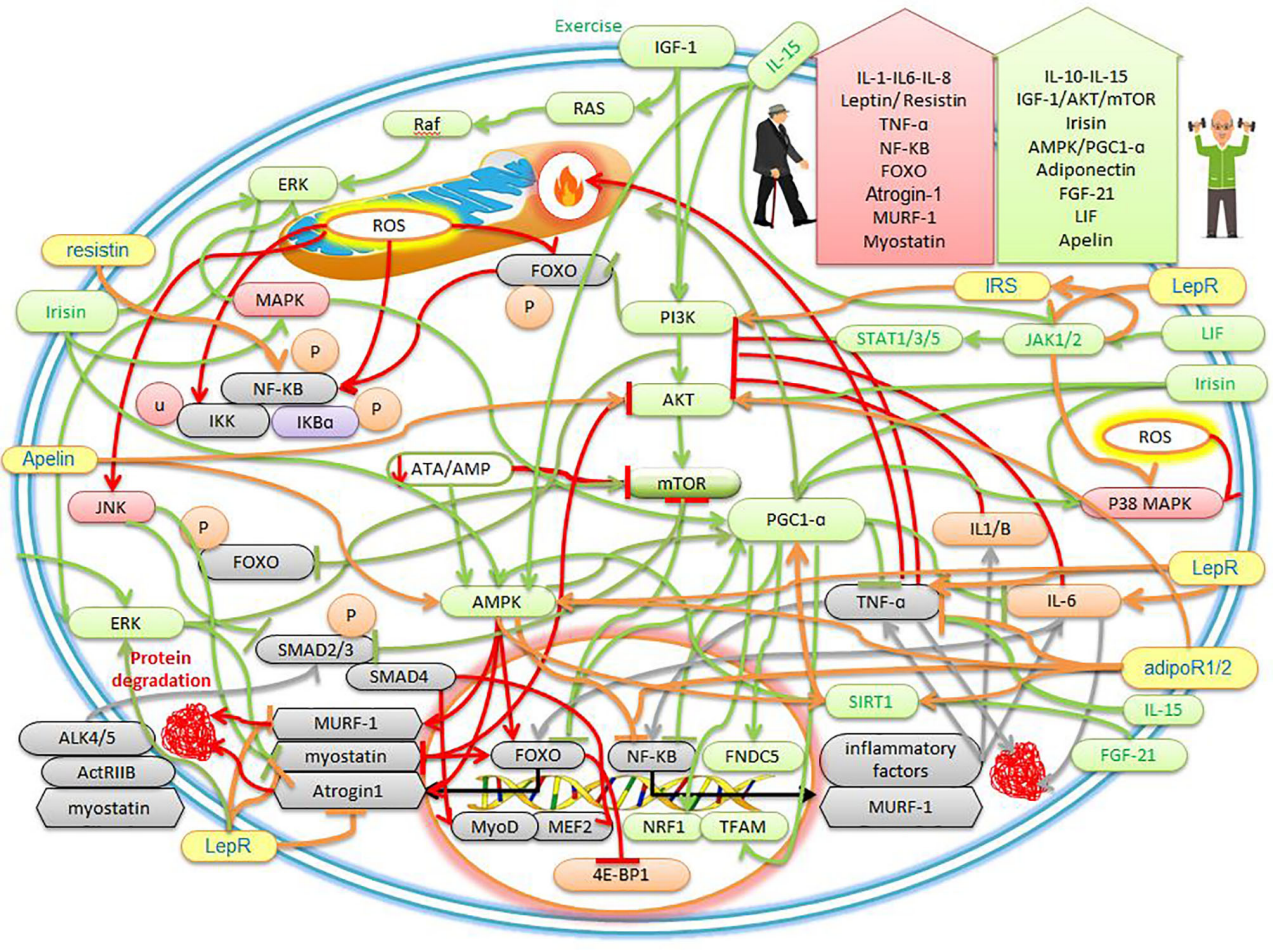
The effects of sarcopenic obesity and exercise on the cellular mechanisms of myokines and adipokines. The description is available in the text.

In humans, IL-15 mRNA is expressed more in type II muscles than in type I muscles, but the IL-15 protein content is expressed in almost all muscles. Studies have shown that IL-15 works through the Janus kinase (JAK)/signal transducer and activator of transcription proteins (STAT) signaling pathway as well as the PI3K/Akt and AMP-activated protein kinase (AMPK) signaling pathway (29) (**Figure 1**). IL-15 is known as an anabolic agent for muscle growth, reducing adipose tissue mass, and stimulating the progression and survival of NK lymphocytes. IL-15 has the anti-apoptotic ability by inhibiting TNF-α pathways (18, 29). In addition, studies have shown that there is a strong association between inflammation, sarcopenia obesity, and pro-inflammatory factors, all of which are thought to be major catabolic mediators in skeletal muscle (30). For example, mitochondrial dysfunction and inflammatory cells lead to increased production of ROS and immune mediators (i.e., cytokines and chemokines) that cause tissue damage at the site of inflammation, cellular aging, and apoptosis (30–32). In the elderly, muscle IL-15 protein and serum levels gradually decrease with age. Decreased plasma IL-15 levels are associated with sarcopenia (13). IL-15 levels are significantly higher in controls compared to sarcopenia (33). One study found IL-15 to be a promising treatment for muscle loss under cachexia and sarcopenia conditioning (34). Serum IL-15 levels increase significantly in centenarians living independently, indicating that high IL-15 expression is one protection against weakness and age-related diseases (13). IL-15 is highly expressed in skeletal muscle and increases in response to exercise for skeletal muscle hypertrophy (13). Human IL-15 genes are positively associated with the response to resistance training (35). Decreased IL-15 levels are reported to be a common mechanism for sarcopenia and obesity, while IL-15 levels are temporarily elevated immediately after resistance and aerobics training (36). IL-15 has the potential to reduce obesity and increase lean mass through the AMPK and Akt pathways, which increase after exercise in humans and rodents. Elevated plasma IL-15 is involved in stimulating the expression of mitochondrial-related factors, such as PPARs and SIRT1, and with increasing circulating IL-15, endurance capacity increases (37, 38). Older and obese mammals are reported to have low blood IL-15 levels, while IL-15 levels are temporarily elevated after resistance and aerobic training. IL-15 can also inhibit fat cell differentiation and reverse dietary obesity (25). In general, with exercise, IL-6-10-15 are secreted as myokines, which can play a role in reducing inflammatory factors, increasing muscle hypertrophy, and protein synthesis through the PI3K/AKT and JAK/STAT pathways. On the other hand, increased lipolysis and decreased adipose tissue occur through increased factors such as PPARs, SIRT1, and AMPK, which can be beneficial for people with sarcopenic obesity (**Figure 1**). However, under inactivity, IL-6 and other inflammatory factors are secreted from adipose tissue, which stimulates sarcopenic obesity and exacerbates muscle atrophy.

## Myostatin

Myostatin is found in large amounts in skeletal muscle (39). Myostatin binds to the active type IIB receptor (ActRIIB) and forms a heterodimer with activin-like kinase 4 (ALK4) or ALK5, which in turn activates Smad2 and Smad3, then forms a complex with Smad4, this complex is subsequently transferred to the nucleus (13, 40) (**Figure 1**). This complex affects transcription factors such as myocyte-specific enhancer factor (MEF2) and myoblast-determining protein (MyoD), which inhibits myoblast proliferation and differentiation (39). In addition, myostatin regulates the activity of paired box 7 (Pax7) to control the process of satellite cell self-renewal. Among myostatin-deficient satellite cells, the number of satellite cells increases due to increased self-renewal and delayed expression of the differentiation gene (myogenin). Thus, myostatin is a strong negative regulator for satellite cell activation. Myostatin also inhibits the Akt/mTOR pathway to suppress skeletal protein synthesis and acts through FOXO-1 to increase skeletal muscle atrophy. Myostatin, as a myokine, is a negative regulator of skeletal muscle growth (13) (**Figure 1**). It was also found that myostatin gene expression can be affected by NF-KB, exercise, aging, and TNF-α. This study found that serum myostatin levels increased with age in the general population (39). myostatin has highest in weak older women and has inversely related to skeletal muscle mass (40). There is evidence that human sarcopenia shows an increase in myostatin and a decrease in Akt phosphorylation efficiency. In this study, myostatin mRNA and protein levels increased by about 2 and 1.4 times. While the efficiency of Akt phosphorylated decreased by about 30%. Thus, human sarcopenia may be associated with decreased activity of IGF-1 and Akt (41). Studies emphasized that myostatin and TNFα are potential candidates for sarcopenia obesity (39, 41, 42). Overexpression of myostatin has been shown to induce strong TNF-α expression. It was also stated that the induction of myostatin expression is mediated *via* the mitogen-dependent pathogen p38 (p38MAPK) and NF-KB pathways through TNF-α (39). In contrast, the deletion of functional mutations in myostatin causes skeletal muscle hyperplasia and hypertrophy (40). exercise studies have shown that simultaneous resistance and endurance training reduce myostatin in sarcopenic older men (43). Exercise after 12 weeks of resistance and endurance training simultaneously increases the levels of irisin and follistatin and reduces the concentration of serum myostatin in obese people (44). One study reports that obese people positively regulate myostatin levels and show an association between sarcopenia, obesity, and myostatin inhibitors, and report the low activity as the cause of increased myostatin (45). It is also shown that sarcopenic obesity is associated with positive regulation of TNF-α, IL-6, and myostatin and a decrease in IL-15, while combined exercise significantly reduces the symptoms of sarcopenic obesity by inhibiting myostatin (25). In sarcopenic obesity, myostatin is regulated positively, and weight loss and adequate protein intake with exercise are recommended as treatment (46). Therefore, as myostatin levels increase with age and persistent inflammation, and decrease with exercise, it seems that exercise can be used as a standard intervention to prevent exacerbation of sarcopenia obesity.

## Insulin-Like Growth Factor-I (IGF-I)

Skeletal muscles secrete insulin-like growth factor-1 (IGF-1) in an autocrine and paracrine manner (1). IGF-1 is an anabolic myokine that increases satellite cell proliferation by increasing cell cycle progression through activating the PI3K/Akt signaling pathway (13). The Ras/Raf/ERK pathway is activated by IGF-I that can increase cell proliferation, differentiation, and survival (1) (**Figure 1**). In addition, IGF-1Ec, called mechanical growth factor (MGF), stimulates the activation, proliferation, and fusion of satellite cells to repair and maintain muscle (13). IGF-I/Akt/mTOR axis inhibits myostatin-dependent pathways such as Smad2/3 in addition to myoblast differentiation and myotube hypertrophy (47) (**Figure 1**). In particular, IGF-1 enhances myogenesis and increases the strength of myofibers (13). Muscle-induced IGF-I controls growth, survival, and differentiation after injury or exercise (1). In addition, decreased serum IGF-1 levels are independently associated with the risk of sarcopenia and increased abdominal visceral fat (5, 13). IGF-1 secretion decreases with age in men approximately 60 years and older, which is associated with sarcopenia, muscle weakness, and upper extremity obesity (48). Therefore, IGF-1 impairment is likely to be associated with the risk of developing sarcopenic obesity (1). IGF-1 plays a therapeutic role in muscle atrophy due to increased protein synthesis (13). In mice with overexpression of IGF-I, decreased expression of age-induced satellite cells in skeletal muscle is reduced. In older mice, overexpression of IGF-I in muscle causes skeletal muscle hypertrophy and regeneration (1). There is evidence that after exercise, plasma levels of inflammatory markers IL-6 and TNF-α decreases, while IGF-1 levels increase (49). When local IGF-I is released, it stimulates satellite cell proliferation, increases muscle amino acid uptake, reduces proteolysis, and facilitates muscle recovery after injury or exercise (13). Clinical studies reported aerobic exercise in people with sarcopenia increases IGF-1 and decrease muscle RING-finger protein-1 (MuRF-1), myostatin, and TNF-α. It was also shown that 8 weeks of aerobic exercise prevent muscle atrophy by increasing the axis of Akt/mTOR/p70S6K since Akt then inhibits the FOXO to suppress protein degradation factors. The study reported that 4 weeks of resistance training inhibits muscle atrophy by regulating MuRF-1 and Atrogin-1 mRNA levels, then reducing ROS and upregulating the IGF-1/Akt/ERK signaling pathway in the soleus muscle (5) (**Figure 1**). Exercise studies have shown that serum IGF-1 levels are higher in the elderly with sarcopenic obesity after resistance training than in the non-exercise group (50). It was reported that home exercise increase IGF-1 in the progressive resistance training group and the aerobic group, while IL-6 decrease in the progressive resistance training group. The researchers concluded that progressive resistance training could improve muscle strength by increasing IGF-1 and decreasing IL-6 (50). Exercise studies showed that at week 8 training, the serum concentration of IGF-1 in the resistance training group is higher in sarcopenic obesity patients than the control group, while the combined training was superior to the endurance and control groups. Muscle strength performance and serum IGF-1 levels were higher than the control group in the training groups, especially in the resistance training group (48). Therefore, the prevailing view is that IGF-1 decreases in obese people with sarcopenia, while it increases with exercise, especially resistance training.

## Irisin

Irisin, as a myokine, is known in muscle as a cleavage product of fibronectin type-III domain-containing protein 5 (FNDC5) and is regulated by PGC1-α. The FNDC5 gene is secreted in human muscles 200 times more than in fat cells (13). Irisin regulates myogenic differentiation, mitochondrial function, and metabolic homeostasis in skeletal muscle. Irisin also increases the expression of PGC-1α, uncoupling protein 1 (UCP1), and other brown fat-related genes in the WAT to stimulate browning and promote energy expenditure (13). One of the most important functions of irisin is to increase temperature and heat and can reduce weight (13). Irisin improves energy expenditure through the MAPK, p38 MAPK, and ERK pathways, and decreases obesity since these three factors converge in the PGC-1α (6, 51) (**Figure 1**). In addition,24-hour treatment with Irisin significantly increases PGC-1α, nuclear respiratory factor 1 (NRF1), and transcription factor A mitochondrial (TFAM), leading to increased mitochondrial rate and oxygen consumption (52). Treatment by irisin significantly upregulates insulin-like growth factor-1 (IGF-1) and decreases myostatin gene expression through the ERK pathway (52, 53). Irisin and the initial expression of FNDC5 have also been shown to be increased in the muscles of myostatin-deficient mice (52). Moreover, myostatin-deficient mice brown adipose tissue increase in white fat stores and are regulated by the AMPK-PGC1α-FNDC5 pathway in skeletal muscle (52) (**Figure 1**). FNDC5 and irisin mRNA secretion is increased during myogenic differentiation in human myocytes (52, 53). Studies have shown that irisin upregulates the IGF-1/Akt/mTOR pathway and increases muscle hypertrophy through muscle protein synthesis (53) (**Figure 1**). In this way, irisin, as a promyogenic factor, induces myogenesis and mitochondrial biogenesis and protects against muscle atrophy. Therefore, increasing the level of irisin can cause cell proliferation (54). Circulating irisin levels in men and women are also negatively correlated with age. In contrast, circulating irisin levels are associated with lean body mass ratio and hand strength in both men and women. In addition, circulating irisin levels are lower in the sarcopenia group than in the normal group (55). Therefore, serum levels of irisin could be used as a biological marker of muscle dysfunction to independently predict the onset of sarcopenia obesity (13, 56, 57). Elderly studies have reported lower levels of irisin in the bloodstream of older people than younger people. In the elderly, it seems the use of irisin to target AMPK-PGC1α-FNDC5 and IGF-1/Akt/mTOR pathways appears to prevent muscle disorders (58). Irisin treatment can cause skeletal muscle hypertrophy, improve muscle strength, and reduce tissue necrosis and fibrosis in the dystrophic rats model. Serum irisin levels are positively associated with increased muscle mass, strength, quadriceps cross-sectional area/body weight, and metabolism (13, 59). Studies have shown that endurance training for 10 weeks increases FNDC5 mRNA levels in skeletal muscle and circulating irisin levels in obese people (51). Resistance training three times a week for 12 weeks in older mice and humans significantly is increased circulating irisin. The researchers also stated that the increase in circulating irisin due to resistance training was strongly associated with improved physical function (58). Postmenopausal women with sarcopenia have been reported to have lower circulating irisin concentrations, while resistance training alone or in combination with aerobic exercise improves muscle size and strength, and these improvements are associated with decreased myostatin and increased serum irisin concentrations (53). One study reported that exercise increased irisin levels in overweight women (44). In human muscle, irisin, IGF-1, FNDC5 (which encodes the irisin precursor) increase after exercise, and irisin is independently associated with sarcopenia (19). The results of exercise studies showed that low plasma irisin is a sign of muscle weakness and atrophy and is associated with total muscle mass. While resistance training in men aged 60-75 years leads to a significant increase in strength gaining and irisin (60). Therefore, higher plasma irisin levels have been described in obese and sarcopenia humans after exercise. Finally, it seems increasing exercise-induced irisin increase mitochondrogenesis, increased brown fat, increased muscle mass, increased body temperature, increased oxygen consumption, increased mitochondrial efficiency, and can be beneficial for people with sarcopenic obesity.

## Fibroblast Growth Factor-21 (FGF-21)

Fibroblast growth factor-21 (FGF-21) is a member of the FGFs family and is mainly secreted in adipose tissue and skeletal muscle (13). Under normal conditions, the expression level of FGF-21 in skeletal muscle is low (61). But muscle FGF-21 is released under conditions such as fasting, exercise, ER stress, mitochondrial myopathy (62). Muscle-specific FGF-21 acts as a vital regulator of muscle growth, inflammation, whole-body metabolism, and premature aging (13). FGF-21 and its receptors such as β-Klotho, FGFR1b, FGFR1c, and FGFR4 are strongly increased in muscle under catabolic conditions (61). In addition, FGF-21 enhances myoblast differentiation and acts through the FGF-21/SIRT1/AMPK/PGC-1α axis to convert anaerobic myofibers to aerobic (11, 13) (**Figure 1**). FGF-21, as an anti-inflammatory agent, stimulates the oxidation of fatty acids, the production of ketone bodies, and inhibits lipogenesis (63). FGF-21 is a major regulator of brown fat differentiation by increasing the expression of UCP1 and PGC-1α and thus generates heat in adipose tissue and skeletal muscle (11, 64). In confirmation of this, a study reported that suppression of FGF-21 prevents white adipose tissue from turning brown. In addition, FGF-21 is usually increased to increase defective mitochondrial function and stress adaptation in skeletal muscle (61). In this regard, FGF-21 has been shown to induce mitophagy by increasing BCL2 interacting protein 3 (Bnip3). Whereas, in the absence of FGF-21, mitophagy reduction is associated with an increase in defective mitochondrial volume (63). Due to the special importance of FGF-21, mice lacking FGF-21 are not able to fully express PGC-1α (65). In this way, FGF-21 has been suggested as one of the markers of mitochondrial dysfunction and aging (61). In skeletal muscle, obese mice lacking FGF-21 positively regulate the expression of atrophic factors such as MuRF1 and Atrogin-1. These factors are associated with increased levels of inflammatory cytokines such as TNFα and MCP-1 and decreased AMPK phosphorylation. While treatment with FGF-21 significantly suppresses TNF-α-induced inflammatory and atrophic responses (13, 66). Muscle-derived FGF-21 plays an important role in aging and obese, and a positive correlation has been found in serum FGF-21 levels and sarcopenia and obese. Thus, sarcopenia and sarcopenic obesity may be associated with FGF-21 disorder (11, 13, 67–69). In this regard, a study identified FGF-21 as potential therapeutic targets associated with PGC-1α for aging and age-related diseases (65). A mild mitochondrial transplant in skeletal muscle increases FGF-21, which in turn leads to healthy aging and longevity. Thus, it seems overexpression of FGF-21 in mice increases longevity (61). It has also been reported that under physiological conditions such as exercise and cold, levels of FGF-21 increase due to muscle contraction and mitochondrial stress (65). FGF-21 gene therapy in obese animals with a high-fat diet leads to weight loss, reduced adipose tissue hypertrophy, reduced inflammation, and fibrosis. In addition, overexpression of FGF-21 prevents age-related weight gain, and this study underscores the potential of FGF-21 gene therapy for the treatment of obesity (13, 64). Studies have reported that after acute exercise, FGF-21 levels increase in plasma, mRNA, and protein levels in muscle. While 3 hours after exercise increases the oxidation of fatty acids (70). In people with obesity, acute exercise increases the concentration of FGF-21 in one hour after exercise (71). After 8 weeks of progressive resistance training in obese mice, levels of FGF-21 and irisin in soleus muscles increase and show a significant relationship with strength gain (72). It has been reported that there is a positive correlation between FGF-21 and irisin with muscle mass (73). Resistance and endurance training have also been shown to increase and decrease FGF-21 and FGF-19 and are beneficial for lipid metabolism (74). In response to exercise, FGF-21 is released from muscle into the bloodstream and prevents some diseases, especially sarcopenia and obesity (75). In general, by increasing energy consumption in exercise, the body increases mitochondria as an energy generator in muscles through FGF-21, irisin, and PGC-1α. Therefore, an increase in FGF-21 in aging seems to be a pathological sign to counteract mitochondrial dysfunction through the mitophagy process. But increasing FGF-21 during exercise is a physiological response to optimizing mitochondria and reducing adipose tissue, converting white to brown fat, and increasing thermogenesis.

## Leukemia Inhibitory Factor (LIF)

Leukemia inhibitory factor (LIF), a myokine belonging to the IL-6 family, is produced by different tissues, such as cardiac and skeletal muscle (4). LIF signaling begins after LIF binding to a specific LIF receptor (LIFR), leading to phosphorylation and activation of Janus kinase (JAK)/signal transducer and activator of transcription 1 (STAT1) and STAT3 in the muscle. LIF also induces the expression of suppressor of cytokine signaling proteins (SOCS), which negatively regulates LIF signaling at the receptor level (76). Furthermore, LIF induces the proliferation of satellite cells by regulating the expression of jun B and c-Myc, and in particular the JAK2-STAT3-PI3k signaling pathway in skeletal muscle. Thus, LIF regulates myoblast proliferation, which acts as an autocrine (13). LIF is a pleiotropic cytokine with increased myoblast survival that has positive effects on myogenesis (77). LIF also stimulates the proliferation of satellite cells (SCs) after sports injuries for muscle regeneration and muscle hypertrophy (4). LIF is involved in increasing muscle glucose uptake, stimulating osteoblast differentiation, and inhibiting fat differentiation and inflammation and performs these effects through the autocrine and paracrine pathways (78). Muscle of LIF-deficient mice reduces regeneration after muscle injury, while local administration of LIF shows regeneration stimulation. LIF has anti-inflammatory properties because LIF downregulates the expression level of TNF-α induced by lipopolysaccharide (LPS), while the LIFR-α antagonism in the muscle regeneration phase increases inflammation and prevents myotubes formation (79). LIF appears to have beneficial effects against muscle atrophy and is a new biomarker and therapeutic target for sarcopenia obesity (13). In one study, it was reported that LIF production and secretion increase with electrical stimulation and overload, and then regulate hypertrophy *via* the PI3K/Akt/mTORC1 axis (80) (**Figure 1**). It was stated that LIF is essential for the hypertrophic response because there is no muscle growth in animals without LIF. It then was claimed that LIF acts specifically on muscle fibers because the administration of LIF for 4 weeks only causes soleus hypertrophy, while extensor digitorum longus muscles had needed a β2-adrenoreceptor agonist to respond to growth (80). This myokine is released by acute aerobic and resistance training. Exercise studies have shown that the secretion of exercise-induced lipolytic myokines (IL-6, irisin, and LIF) stimulates thermogenesis for converting white to brown fat (4). Interval exercise increases LIF/LIFR expression in muscle and stimulates the STAT3 signal to reverse muscle atrophy in mice. In addition, LIF transgenic leukocytes can reduce fibrosis and play a potential therapeutic role in improving muscular dystrophy and sarcopenic obesity (13). Studies have reported that LIF mRNA increase after ergometer cycling and endurance training. It was also stated that LIF has the potential to treat muscular diseases such as muscular dystrophy due to increased myoblast survival (75). In humans, one session of resistance training overexpresses LIF mRNA, while not altering circulating LIF levels (80). Studies have shown that LIF mRNA levels in muscles increased 9-fold 6 hours after resistance training while returning to pre-training levels 24 hours after training. LIF in plasma is also unchanged, indicating a local rather than systemic effect of LIF on exercise response. However, the PI3K, Akt, and mTORC1 molecules contribute to LIF signaling in exercise, as the chemical inhibition of each independently is sufficient to downregulate LIF. JunB and c-Myc transcription factors, which increase myoblast proliferation, increase in skeletal muscle after resistance training (81). Measuring LIF protein levels after exercise is difficult because the half-life of LIF is about 6-8 minutes in serum. Therefore, expression and secretion of LIF protein after exercise have not been reported (78). Finally, it seems that exercise by increasing LIF expression can affect people with sarcopenic obesity by growing muscle cells, reducing fat cell differentiation, and stimulating osteoblasts, and can be used as a standard golden intervention.

## Adipokines Affecting Sarcopenic Obesity

In people with sarcopenic obesity, muscle mass decreases quantitatively and qualitatively, and fat mass increases. In addition to myokines, adipokines also play an important role in sarcopenic obesity (46). Adipokines such as leptin, resistin, adiponectin, and apelin can also regulate muscle metabolism (4). Since many disorders of sarcopenic obesity are caused by the secretion of adipokines, we intend to study adipokines related to sarcopenic obesity to be able to better manage this disease to increase the quality of life.

## Leptin

Leptin is mainly secreted from adipose tissue and plays a key role in reducing food intake, increasing energy intake, raising body temperature, and lowering blood sugar. Leptin performs its functions through the leptin receptor (LepR) and the leptin receptor consists of six isoforms: LepRa, LepRb, LepRc, LepRd, LepRe, and LepRf. LepRb appears to regulate most leptin functions. After the binding of leptin to this receptor, activation of tyrosine kinase Janus kinase 2 (JAK2) is performed. Activated JAK2 then participates in the activation of the MAPK and ERK pathways and mediates energy homeostasis. STAT 5 and STAT3 are phosphorylated by JAK2 and amplify target transcription factors (**Figure 1**). Leptin has proinflammatory activity by increasing the production of TNF-α, IL-6, and IL-12 by monocytes (82). Leptin may also act by increasing IGF-I circulation. Leptin indirectly regulates muscle regeneration by suppressing miR-489, an inhibitor of muscle satellite cells. Treatment by leptin in aging mice improves muscle mass and muscle fiber size by reducing the expression of myostatin, 1-muscle ring protein (MuRF1), and F-box muscle atrophy (MAFbx) (1). Therefore, leptin will be useful for both increasing muscle mass and regenerating and repairing muscles. These parameters are related to muscle strength in people with sarcopenia (1). Leptin reduces muscle triacylglycerol (TG) by rapidly activating AMPK, increasing hydrolysis, and oxidation of fatty acids (83). Serum leptin levels reflect total body fat mass (65). Serum leptin levels are usually positively correlated with body mass index (BMI). Leptin levels have positively correlated with longevity in people 100 years old, and the ratio of leptin to adiponectin is positively correlated with muscle strength in older adults (84). Increased leptin due to increased fat in aging may lead to leptin resistance and reduce the oxidation of fatty acids in muscle and lead to fat deposition in other organs such as liver, heart, and muscles, which in turn reduces muscle quality in people with sarcopenic obesity (18). In obese people, despite the increase in leptin levels, there is a lack of positive function of leptin in muscle due to leptin resistance. Leptin receptors are highly expressed in muscle tissue, and a lack of leptin-sensitive receptors, especially in skeletal muscle, causes muscle atrophy. Therefore, leptin resistance may worsen sarcopenia in obese and elderly patients (1). Animal studies have shown that the long form of the leptin receptor exhibits features of sarcopenic obesity such as muscle atrophy, hyperphagia, hyperinsulinemia, and hyperleptinemia (85). Research has shown that patients with sarcopenic obesity have higher serum leptin levels than those with non- sarcopenic obesity. In addition, they show poor grip strength and physical function. Thus, high levels of leptin probably due to leptin resistance are also negatively associated with physical function (86). Mammalian with sarcopenia obesity upregulates TNF-α, IL6, leptin, and myostatin, and downregulates adiponectin and IL-15 (25). According to current evidence, strength and aerobics training combined with nutrition is the most promising approach to sarcopenic obesity. Exercise and diet have been reported to improve gait speed and leptin (87). One study reported that exercise affects the balance of pro-inflammatory cytokines such as leptin (88). Sarcopenic obesity markers such as IGF-1, leptin, and adiponectin improve significantly after 16 weeks of endurance and resistance exercise compared to usual care (10, 25). In addition, exercise improves leptin sensitivity in the peripheral tissues of obese mice (89). In older mice, an increase in leptin is associated with high inflammation and muscle atrophy, while exercise interventions are associated with a decrease in adipose tissue mass and a decrease in serum leptin concentration due to muscle tissue sensitivity to leptin (83). Finally, according to the above studies, exercise appears can lead to decreasing inflammation, decreasing muscle atrophy, increasing muscle hypertrophy, and decreasing adipose tissue by improving leptin resistance. Then active lifestyle increases the life expectancy of people with sarcopenic obesity.

## Adiponectin

Another important hormone produced by adipose tissue is adiponectin (1). Adiponectin signals through adiponectin 1 and 2 receptors (adipoR1 and adipoR2) (3), which are abundantly expressed in muscles and myotubes (82). AdipoR1 is mainly expressed in skeletal muscle and the binding of adiponectin to AdipoR1 activates the AMPK-SIRT1-PGC-1α axis (90) (**Figure 1**). Adiponectin also reduces inflammation by inhibiting the secretion of TNF-α and IL-γ and increasing the production of IL-10 and IL-1 receptor antagonists from monocytes and macrophages (6). There is evidence that adiponectin for AMPK activation and NF-KB inhibition is associated with a decrease in inflammatory factors (TNF-α and IFN-Y) and an increase in anti-inflammatory factors (IL-10 and IL-1Ra) (82). This adipokine has key roles such as regulating energy homeostasis, strong anti-inflammatory effects, increasing myogenesis. In addition, this hormone has a protective effect on muscle protein degradation by regulating the IRS-1/Akt signaling pathway (1). Adiponectin appears to regulate several transcription factors for muscle regeneration, such as Myf5, MyoD, myogenin, and Mrf4 (90). For example, the increase in Myf5 by adiponectin in skeletal muscle provides activation of satellite cells and their commitment to regeneration. In addition, adiponectin induces MyoD expression to allow myoblast proliferation and differentiation (90). Adiponectin also stimulates AMPK-induced autophagy in myoblasts and promotes their survival. Adiponectin activates the expression of two key muscle differentiation factors, myogenin and Mrf4 (90). Thus, adiponectin can reverse sarcopenia by suppressing anti-atrophy proteins and stimulating myogenic proteins (3). Some studies have reported low levels of adiponectin in people with sarcopenia, and an inverse relationship between adiponectin and the risk of sarcopenia (1). Adiponectin levels have been shown to decrease with age and obesity (82). There is a strong negative relationship between circulating adiponectin concentration and fat mass (25). Thus, in sarcopenic obesity, serum levels of adiponectin decrease (6). There is evidence that chronic exercise-induced muscle improvements are reduced by suppressing adiponectin or AdipoR1. Similarly, AdipoR1-deficient mice reduce PGC-1α expression and activity, mitochondrial biogenesis and function, type 1 oxidative myofibers, endurance training capacity, and cellular clearance (90). Studies show that adiponectin is significantly increased in people with sarcopenia under positive lifestyle intervention (whey supplementation with resistance training). Because, high levels of adiponectin have been observed in centenarians, due to a compensatory response to maintain metabolic homeostasis, reduce inflammation, reduce oxidative stress, and protect against catabolic conditions such as sarcopenia (1). In addition, weight loss due to exercise increases plasma adiponectin levels in rodents and humans while adiponectin decreases in the circulation of old and obese people (82, 90). An increase in plasma adiponectin has also been observed in moderate to high aerobic exercise. Thus, higher levels of adiponectin have been reported in parallel with most physical activity. Exercise for 4 months reduces impaired regeneration and muscle function in older mice (90). Exercise-adiponectin released activates fatty acid oxidation and glucose uptake *via* the AMPK pathway, which is reduced in sarcopenic obesity (82). Exercise stimulates muscle stem cells, improving muscle regeneration capacity by activating adiponectin/AdipoR1 in aging-prone mice (91). In addition, endurance training can ameliorate age-related disorders in muscle stem cell regeneration and muscle metabolic changes through the AMPK-dependent mechanism regulated by the adiponectin/adiponectin receptor axis (91). Therefore, it is possible that exercise can increase adiponectin in sarcopenic obesity, which in turn leads to increased muscle hypertrophy, decreased inflammation, and increased fat oxidation. So exercise can be considered a non-invasive golden intervention.

## Resistin

Resistin is released by adipocytes, myocytes, and leukocytes. Resistin secretes TNF-α and IL-6 from human blood leukocytes, and in contrast, expression of resistin is stimulated by IL-1, TNF-α, and IL-6, indicating a defective pro-inflammatory loop (36) (**Figure 1**). Increased secretion of resistin by subcutaneous adipose tissue disrupts myotube and nuclear fusion by activating the classical NF-KB pathway (92). In muscle, resistin inhibits myogenic myoblast differentiation (1). While reducing resistin myogenesis regenerates aging muscle. Inhibition of the classical NF-KB pathway in myoblasts protects against the harmful effect of resistin on myogenesis (92). Resistin RNA is more expressed in visceral fat than non-abdominal subcutaneous fat and serum resistin is higher in obese people (36). The concentration of resistin is significantly higher in obese elderly people than in normal-weight elderly people (92). Visceral adipose tissue releases catabolic adipokines such as IL-6, TNF-a, and resistin, which play a key role in protein catabolism in people with sarcopenic obesity (93). In addition, plasma resistin levels increase in the elderly compared to the young and are inversely related to muscle strength. Plasma resistin concentrations are inversely related to quadriceps muscle torque in people aged 69 to 81 years (92). In the elderly, the resistin/IGF-I ratio increases and is associated with decreased quadriceps strength. Resistin acts on the joints *via* NF-KB, which increases with aging (1). Decreasing the resistin of obese people has been shown to improve myogenesis (92). Exercise studies have shown that 12 weeks of resistance training reduces plasma leptin and resistin levels, and concluded that long-term resistance training improves sarcopenia in people by reducing inflammatory markers and reducing adipose tissue (94). In individuals, levels of IL-6, leptin, and resistin show an increase in obesity and are inversely associated with low inflammation but can return with exercise (4). Aerobic exercise five days a week for 12 weeks in sedentary, overweight women increases IL-15 levels and decreases resistin (95). One study found that exercise increase and decrease adiponectin and resistin levels, respectively. The data show that without weight loss, exercise alone does not improve resistin (96). In one exercise study, resistin concentrations decreased after prolonged exercise and were associated with triglyceride concentrations (97). Exercise study showed that 6 weeks of endurance exercise reduces chemerin and resistin, which indicates a reduction in inflammation (98). In one study, a resistance and aerobic training program for 8 weeks significantly increased apelin levels and decreased plasma resistin, insulin, fasting glucose, and insulin resistance index (99). Since there are few studies on exercise, resistin, and sarcopenia obesity, more studies are needed. However, according to the above studies, exercise appears can reduce the NF-KB signaling pathway, protein degradation, inflammation, and adipose tissue by reducing resistin. In contrast, exercise can improve muscle strength and hypertrophy by reducing factors that impair muscle regeneration, such as resistin.

## Apelin

Apelin and the receptor of apelin are expressed in many tissues, such as adipose tissue and muscle (80). Aplin binds to the APJ receptor, a member of the G protein-coupled receptor family (100). Applin activates endothelial nitric oxide synthase (eNOS), which is known to stimulate glucose transport in endothelial cells and capillaries formation in skeletal muscle (100). Aplin stimulates the phosphorylation of AMPK and acetyl-CoA carboxylase (ACC) and interacts with the insulin signaling pathway (100). Thus, apelin is involved in regulating glucose and fat metabolism as well as insulin sensitivity (80). Apelin stimulates mitochondrial biogenesis and protein synthesis by activating AMPK, AKT, and P70S6K in individuals with sarcopenia (101) (**Figure 1**). Apelin enhances autophagy, and the anti-inflammatory effects of muscle, and enhances the regeneration of satellite cells (102). While removal of the muscular apelin leads to muscle atrophy, decreased strength, and decreased exercise performance on the treadmill, all of which are remedied with daily apelin treatment (80). In mice without apelin, muscle function abnormalities worsen with age. Studies have shown that downregulation of apelin accelerates the onset and progression of aging. For this reason, in the elderly, apelin has been suggested as a primary marker of sarcopenia (103). In people with sarcopenia, the level of apelin and the synthesis of apelin in skeletal muscle decreases significantly with age and is associated with decreased strength (104). Apelin increases with the contraction of human myotubes and after muscle contractions in mice (80). During skeletal muscle regeneration, apelin is secreted along with myogenic progenitor cells (80). Local apelin secretion increases muscle endurance by increasing the number of mitochondria and activating AMPK (80). After apelin injection, skeletal muscle capacity and myofiber hypertrophy are improved, protein synthesis is enhanced, muscle cell regeneration is increased in older mice, and proteolysis in myotubes is inhibited (104). Apelin supplementation has synergistic effects on endurance training and improves fatigue resistance. Overexpression of the muscular apelin, in parallel with the effects of exercise, increases muscle mass (80). One study reported that obese older women increased their apelin levels after walking twice a week for 12 weeks, and an increase in apelin concentration was associated with further motor function improvement (105). There is evidence that pregnant women increase apelin levels under exercise conditions, which in turn leads to increased PR domain-containing zinc finger protein 16 (Prdm16) promoter DNA demethylation, thermogenesis, and increased brown fat. Apelin consumption by the pregnant mother mimics the beneficial effects of exercise on fetal brown fat growth, oxidative phosphorylation, mitochondrial activity, and inhibits the process of lipid synthesis and differentiation of white fat cells in fetal brown fat. The researchers stated that apelin significantly activates thermogenesis through AMPK activation (106). In addition, exercise-induced apelin secretion reverses age-related muscle loss in people with sarcopenia and enhances mitochondrial biogenesis and protein synthesis of muscle fibers (106). One study found that with age, secretion of apelin, decorin, IGF-1, IL-15, and irisin decreased, while IL-6 and myostatin increased. The study stated that aerobic exercise upregulates apelin, IL-15, IL-6, irisin, while anaerobic exercise increases BMP-7, decorin, IGF-1, IL-15, IL-6 (103). Moreover, after aerobic exercise for 8 weeks, a significant increase in apelin expression and secretion was reported. Apelin mRNA expression in mice increased by approximately 40% after 9 weeks of swimming training. Obese men increased apelin mRNA expression 3.3-fold after 8 weeks of aerobic exercise (running and cycling) (103). In addition, increasing apelin levels in the elderly have been reported to increase hypertrophy and help improve motor function. Apelin has also been shown to be more released in athletes than in non-athletes after one session of acute resistance training (107). Generally, apelin as an exercise-induced factor has an anti-sarcopenic obesity function by targeting satellite cells and fat cells (108). Therefore, according to the above studies, exercise-induced apelin secretion appears can cause muscle regeneration, muscle hypertrophy, increased muscle strength, increased mitochondria, decreased muscle atrophy, increased brown fat, decreased white fat synthesis, increased thermogenesis, and decreased sarcopenic obesity.

## Effects of Exercise and Sarcopenia Obesity on Protein Degradation Through FOXO as Myostatin Regulator

Another factor that can affect sarcopenia-related muscle atrophy is forkhead box O (FOXO). FOXO is one of the most important regulators of atrophy that stimulates the expression of many atrophy-related genes (109). Mammalian cells include three members of this family: FOXO1, FOXO3 (FKHR L1), and FOXO4 (AFX) (110). Scientific studies have shown that many of the major transcription factors regulating atrophy-related genes converge on FOXOs (FOXO1, 3, and 4). For example, 96% and 48% of atrophy genes are monitored by FOXO during suspension and denervation, respectively (109). Dephosphorylation of FOXOs stimulates nuclear entry, growth suppression, and apoptosis. Studies have shown that FOXO1 is activated in all muscle atrophies (110). In addition, at least half of the atrogenes need FOXOs to regulate their up or down pathways. FOXO-dependent atrogenes include E3 ubiquitin ligases Atrogin-1 [also known as muscle atrophy F-box (MAFbx)], myostatin, muscle RING finger 1 (MURF-1), muscle ubiquitin ligase of SCF complex in atrophy-1 (MUSA1), specific for muscle atrophy and regulated by transcription (SMART) (109, 110) (**Figure 1**). Further Atrogin-1/MAFbx transcription is controlled by FOXO, whereas MuRF-1 transcription is mediated by NF-KB activation (109). Studies have shown that activation of FOXO1 causes transcription of Atrogin-1 promoter and reduction in fiber size. The fibers overexpressing FOXO3 increase Atrogin-1 mRNA levels. In many cells, FOXO transcription delays cell cycle development and activates apoptosis (110). In sarcopenic obesity, an increase in perimuscular adipose tissue (PMAT) increases the nuclear transfer of FOXOs transcription factors and regulates Atrogin1 and MuRF1, leading to proteolysis in muscle tissue. In addition, PMAT levels are associated with the severity of muscle atrophy (6). Studies have also shown the presence of functional binding sites for FOXO1 in the myostatin promoter, suggesting stimulation of myostatin gene expression through the FOXO1 transcription factor (111). Two ubiquitin E3 ligases, such as Atrogin-1 and MuRF-1, are proteins for skeletal muscle atrophy conditions and are associated with apoptosis in skeletal muscle aging. Increased apoptosis can activate Atrogin-1 and MuRF1 ligases, increasing protein degradation and exacerbating age-related skeletal muscle loss. Therefore, one of the characteristics of older mice is an increase in Atrogin-1, MuRF1, and myostatin (112). Scientific studies have shown that mitochondrial damage and dysfunction due to inactivity can increase proteolysis *via* the AMPK-FOXO3 axis (111). AMPK modulates FOXO3 transcription activity for muscle atrophy by activating the ubiquitin-proteasome pathway. ROS production by defective organelles such as mitochondria causes muscle atrophy by activating the FOXO signaling pathway (113). In response to inflammation, FOXO1 activity increases and NF-KB transcriptional activity promotes downstream genes. Then activated NF-KB with FOXO1 acts synergistically, increasing the expression of pro-inflammatory factors such as IL-1β (114). On the other hand, inflammation is associated with FOXO activation and myostatin overexpression, which in turn can cause NF-KB-independent muscle atrophy (14). One study found that the expression of SMAD and FOXO increased myostatin promoter activity and inhibited myoblast differentiation (115).

Specific inhibition of FOXOs in muscles protects against atrophy. Inhibition of FOXO3 and NF-KB transcriptional activity by PGC-1α and β reduces protein degradation (113). However, PGC-1α is identified as a direct transcription target of FOXO1 (116). Inhibition of FOXO family members is essential for muscle differentiation with phosphorylation (110). In addition, FOXO1 is downregulated by the PI3k/Akt pathway, because the IGF1-AKT-mTOR axis blocks FOXOs transcription factors and protein degradation pathways (**Figure 1**). Protein kinase B (PKB)/Akt is a serine/threonine kinase that signals through the downstream pathway of growth factor receptors by activating PI3K, whose activity can be mediated by IGF-1 receptor signaling, nutrients, and muscle contraction. Akt plays a variety of roles that may be important in sarcopenia. These roles include suppressing apoptosis and protein degradation in skeletal muscle by promoting phosphorylation and inactivating the pre-apoptotic transcription factors such as FOXO and Bad, thus inhibiting the expression of atrophy-related genes such as Atrogin-1 and MuRF-1. Akt phosphorylates FOXOs transcription factors to inhibit their translocation to the nucleus (117), exports FOXO1 from the nucleus, and further reduces gene transcription (114). But TNFα, IL-1B, IL-1, and IL-6 directly inhibit PI3K/Akt activity and thus eliminate their inhibition of FOXO. IL-6 has also been shown to reduce skeletal muscle by signaling its receptor, which activates FOXO3 (14) (**Figure 1**). However, when Akt is inhibited in aging muscles, FOXO translocation occurs, increasing the expression of atrophy-related genes such as Atrogin-1 and MuRF-1. FOXO1 then inhibits the function of anabolic pathways in skeletal muscle by reducing the phosphorylation of translation suppressor protein 4E-BP1 and by impairing mTOR and Raptor signaling (117). One study reported an increase in the expression of FOXO1 mRNA levels in aging muscles, and the aging muscle nuclei had more FOXO than the young muscle nuclei (117). Moreover, Atrogin-1 mRNA increased in aged rats and FOXO3A has also been reported among sarcopenia-promoting proteins (117). Adipose tissue around muscle also increases FOXO transport and stimulates its targets such as Atrogin1 and MuRF1 for proteolysis and positively regulates muscle cell aging. Thus, increased proteolysis and aging of muscle cells accelerate muscle atrophy caused by aging and obesity (118).

Studies have shown that acute exercise can increase FOXO1 phosphorylation (119). In people with sarcopenia, exercise increases IGF-1/PI3K/AKT levels and subsequent mTOR activation to induce protein synthesis, while simultaneously suppressing FOXO signaling (120–122). Furthermore, the mediators of muscle loss that exercise may target during aging are myostatin and FOXO3a, as these factors have been reported to decrease after aerobic exercise. One-session eccentric training reduces Atrogin-1 and FOXO3a mRNA levels compared to baseline at 3 and 5 h post-exercise. Concentric training also reduces FOXO1 and FOXO3a proteins compared to baseline. Finally, eight weeks of training induce hypertrophy by reducing nuclear FOXO1 protein levels (123). Thus, aerobic exercise in aging may inhibit FOXO1 activity by increasing FOXO1 phosphorylation and limiting acetylation (114). The data from one study suggest that expression levels of FBXO32 and FOXO1 are higher in women than in men, suggesting that women may have high reserves of FBXO32 and FOXO1. Data show increased expression of FBXO32 and FOXO1 in men compared to women after 3 months of exercise, while the acute increase FBXO32 and FOXO1 returned to baseline after 6 months of training. It is also shown that baseline levels of FOXO3 mRNA are higher in aging women (124). In the muscle atrophy model, weight-bearing exercise significantly activates Akt and mTOR expression and downregulates myostatin and its receptor, ActRIIB, and FOXO1 expression (125). Therefore, the Akt/mTOR and FOXO1/myostatin signaling pathways may be the key to protein synthesis improvement in sarcopenia (125, 126). Studies showed when a muscle is exposed to mechanical stress, c-Jun N-terminal kinases (JNK) initiate muscle growth by inhibiting SMAD and myostatin. JNK inhibits SMAD2/3 nuclear translocation and myostatin-induced transcriptional activity. Therefore, SMAD2 phosphorylation has an inhibitory effect on myostatin activity. This study showed that resistance exercise activates JNK/SMAD signaling for overload-induced muscle hypertrophy in aging mammalian (127) (**Figure 1**). In addition, SMAD2/3 mRNA and protein expression also decreased significantly after 6 weeks of weight-bearing training. These results suggest that running obese rats with body weight may reduce the ActRIIB-SMAD2/3 axis (128). It has also been shown that aerobic and resistance training improves performance by reducing oxidative stress and suppressing SMAD2/3 signaling (129, 130). In addition, the increase in age-related TNF-α protein in gastrocnemius muscle decreases with treadmill exercise, indicating that physical activity limits the systemic inflammatory response in aging mice (30). Aerobic exercise has been reported to increase PI3K/Akt activity in obese diabetic rats while inhibiting the FOXO1/NF-kB/NLRP3 inflammatory axis (114). In general, exercise appears can block FOXO and NF-KB pathways by activating the mechanisms of Akt, PGC1-α, and JNK, and prevent the degradation of protein by suppressing MuRF-1, myostatin, SMAD2/3, and Atrogin-1, and instead activate protein synthesis and prevent the progression of sarcopenia obesity.

## Effects of Exercise and Sarcopenic Obesity on Protein Degradation Through NF-KB as a Downstream Agent of Inflammatory Adipokines

Increased inflammatory factors such as IL-6, resistin, TNF-α, and ROS production are also associated with NF-KB signaling and the ubiquitin-proteasome system in skeletal muscle, both of which may be involved in the pathogenesis of sarcopenia (5). NF-KB is a protein complex and multifunctional regulator of DNA transcription, immune function, inflammation, cell survival, and proliferation responses (122). All NF-KB family members are expressed in skeletal muscle (122). NF-KB activity appears to directly regulate MyoD, a myogenic transcription factor also known as myoblast determination protein 1, and possibly other molecules such as MuRF1 during atrophy (117). In the inactive state, NF-KB is sequestered by I-kappaB alpha (IκBα) inhibitory proteins in the cytoplasm (131). The main functions of the IkB protein include preventing the transfer of NF-KB to the nucleus, preventing the binding of NF-KB to DNA, gradual separation of DNA and NF-KB complexes (31). In the active state, the IκB kinase complex (IKK) phosphorylates IκBα (131). IKK is an enzyme complex that is associated with increased cellular inflammation and activated IkK leads to a decrease in NF-KB in the cytosol. Stimulation of IKK regulates ubiquitination and then proteolysis, leading to the binding of NF-KB (122). Following phosphorylation, IKBα is polyubiquitinated and degraded by the proteasome, enabling NF-KB nuclear translocation and transcriptional activity (132), leading to upregulation of atrophy-related pro-inflammatory genes (133). NF-KB directly alters the production of more than 150 genes, including genes encoding cytokines, regulators of oxidation status, acute-phase reaction, cachexia, and disuse atrophy and apoptosis. The NF-KB/Rel family consists of five members such as p50, p52, p65 (RelA), RelB, and c-Rel. Two of these proteins must be dimerized to facilitate NF-KB binding to DNA and regulate gene expression. Recent evidence suggests that the p50-p65 heterodimer is responsible for most of the NF-KB activity in skeletal muscle. However, NF-KB is the main regulator of immunity and inflammation and regulates the effect of inflammatory factors, in particular, TNF-α and IL-6 on muscle degradation (131). Interestingly, the NF-KB activation pathway is more prominent in the muscles of older animals than in young ones (132). The researchers found that the concentration of NF-KB protein in older human muscles was four-fold higher than in young muscles. This increase in concentration is associated with anabolic signaling defects that lead to muscle wasting and aging. They also showed that proteins (IKKγ, IκBα, and p65) increase with age in the soleus muscle (117). In sarcopenic obesity, IKK levels also increase (6). In addition, it is has been shown excessive expression of muscle IKK in transgenic mice leads to severe muscle atrophy (131).

Exercise studies show an increase in NF-KB inhibitory proteins IκBα after a moderate exercise program. In this way, exercise reduces the activation of the NF-KB pathway and decreases the levels of IL-6, IL-1B, and TNF-α mRNA in obese mice (133, 134). In aging mice with muscle NF-κB knockout, physical function increases, and more resistance to atrophy is shown (135). In addition, regular exercise suppresses high levels of NF-KB in aging, and acute fatiguing exercise could reduce NF-KB activity in human muscles (135). In this regard, 10 weeks of endurance training in the inflammatory condition significantly reduce NF-KB protein and expression of Atrogin-1 and MuRF-1 genes compared to the control group. Therefore, endurance training seems to affect the atrophy mechanisms of NF-KB/Atrogin-1/MuRF-1 (136, 137). Exercise has been shown to reduce serum leptin, IL-6, TNFα, and resistin levels in obese mice and inhibit the MuRF-1 and NF-κB-associated pathway. Exercise increases the SIRT1-AMPKα-PGC1α pathway, the mitochondrial complex IV and I-V, and genes related to mitochondrial biogenesis such as Nrf1 and Tfam. Finally, exercise prevents the reduction of muscle mass and cross-section of the muscle (133). There is also evidence that there are two binding sites for NF-KB in the myostatin gene, which is activated by ROS under hypoxic conditions, but exercise related-muscle hypertrophy is associated with a decrease in myostatin and NF-KB (138). Thus, the above studies have shown that sarcopenic obesity increases inflammatory factors such as IL-6, TNF-α, and resistin, which in turn lead to muscle atrophy through the myostatin, NF-KB, and MURF-1 pathways. While this process can be stopped through adaptation to exercise. Therefore, appears physical activity can be recommended as a golden intervention to fight sarcopenic obesity.

## The Effect of Resistance and Endurance Training on Sarcopenic Obesity

**Figure 2:**
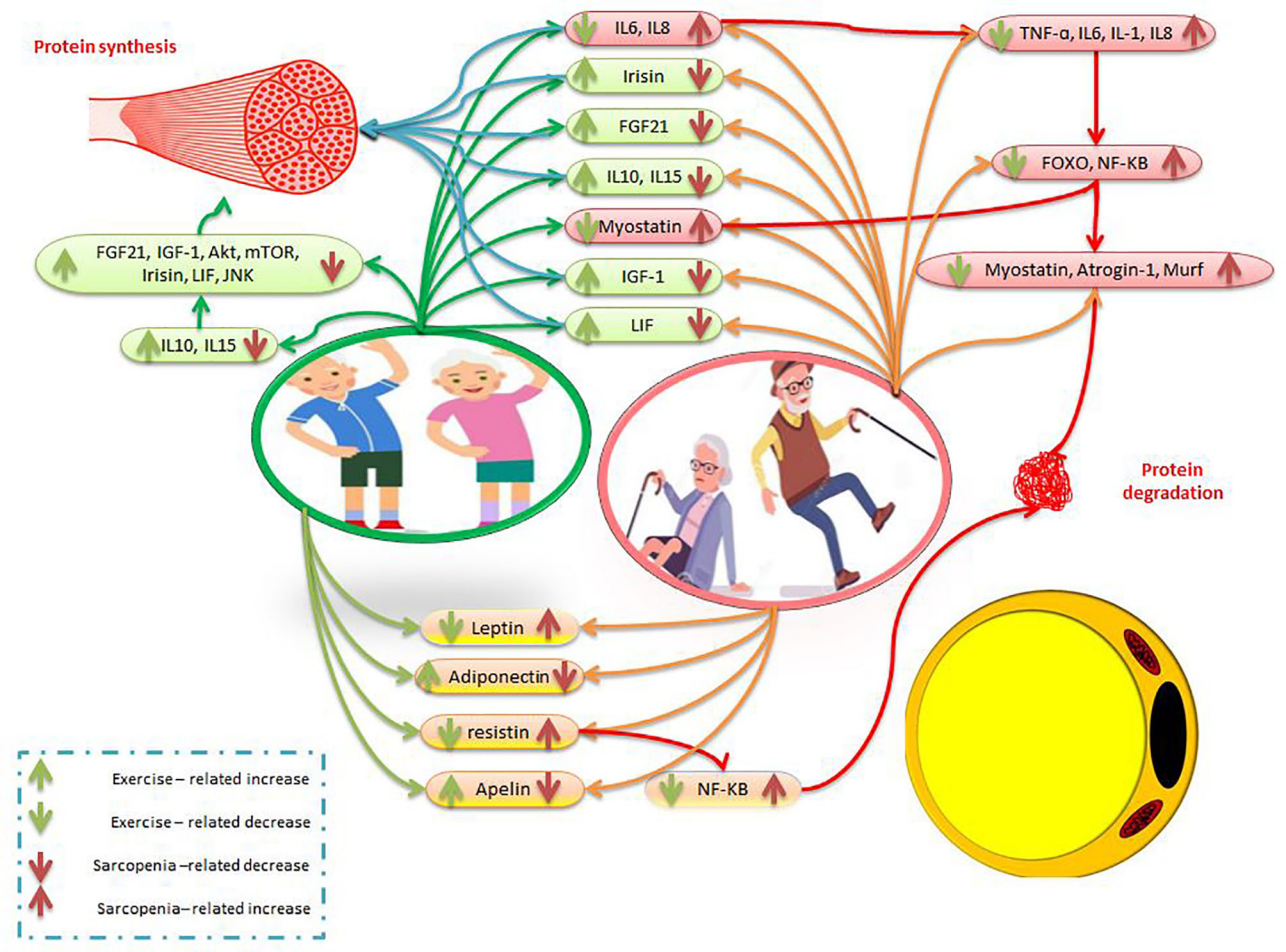
The effects of exercise and sarcopenia obesity on myokines and adipokines. The description is available in the text.

Resistance exercise is one of the most effective non-invasive ways to deal with decreased muscle mass and strength, increased adipose mass in older people with sarcopenic obesity (139). Regular exercise by increasing IGF-1 expression as a myokine can reduce the symptoms of sarcopenic obesity and the risk of falls, and increase muscle strength and hypertrophy (140). While older muscles may reduce IGF-I induction and be resistant to IGF-I, resistance exercise reverses this process and protects against sarcopenic obesity by overexpressing IGF-I muscles (141). Another study found that resistance training after 12 weeks by increasing IGF-1 is an effective tool for muscle hypertrophy and reduced training-related adaptations due to aging (142). In aging people, exercise through mechanical overload increases IGF-1 and mTOR levels to induce protein synthesis, activate satellite cells, and reduce muscle fat (143, 144). In aging women, decreased FOXO3A and myostatin RNA are associated with exercise-induced muscle hypertrophy (145). In addition, one session of intense endurance and heavy strength training leads to a temporary increase in plasma irisin concentration in obese people. This study stated that Intense endurance training and strength training also significantly increase PGC-1α expression, which is involved in controlling FNDC5 transcription (51, 146). In obese people, an increase in circulating FGF-21 levels is observed after exercise to regulate energy metabolism by stimulating glucose and lipid oxidation (147, 148). In people with sarcopenia, interval training has been shown to increase LIF and LIF receptor (LIFR) expression and STAT3 phosphorylation in gastrocnemius muscles, leading to decreased apoptosis and increased cell proliferation. There was also a significant negative correlation between skeletal muscle atrophy and LIF expression. Thus, interval training reverses skeletal muscle atrophy by reducing apoptosis and repairing cell proliferation in the gastrocnemius muscle (149, 150). On the other, one study exposed 68-year-old men to low-, medium-, and high-intensity strength training. In this study, leptin levels decreased in all interventions, while adiponectin increased only in medium and high-intensity resistance training. During the non-training period, training-related changes were maintained only in the high-intensity resistance training group (151). Older postmenopausal women also participated in a four-month strength training program with two training sessions per week. After 16 weeks of training, leptin and resistin levels were reported significantly lower than baseline levels. The researchers concluded that strength training is effective in improving upper and lower limb strength in older women (152). In another study, 63-year-old sedentary women participated in 16 weeks of resistance training. Interleukin-6, leptin, and resistin decreased after exercise compared to baseline levels (153). In addition, in an animal study, the protein levels of irisin, IL15, LIF, and BDNF in the soleus muscle of aging mice increased significantly after 12 weeks of resistance training (58). In aging men, moderate-intensity endurance and resistance training, myokines such as LIF, IL-4, IL-6, IL-7, IL-15, decorin, apelin receptor, and irisin are upregulated in muscles (107, 154) (**Figure 2**). It was also emphasized that 4 weeks of exercise stimulates muscle apelin and then plasma apelin so that decreased muscle apelin production due to aging can be reversed by chronic exercise (101). Contraction-induced muscle apelin can help older people by affecting mature muscle fibers, the process of cell renewal, muscle regeneration, and satellite cells differentiation (101). There is evidence that after one year of resistance training, IKKB mRNA, TNF-α mRNA, and IL-1β mRNA levels in the old male group significantly decrease compared to baseline levels (154). Overall, strength training increases interleukins 6, 10, and 15, IGF-1, irisin, FGF-21, LIF, adiponectin, and apelin, while decreasing myostatin, FOXO, leptin, resistin, and NF-KB (**Figure 2**). Accordingly, strength training in people with sarcopenic obesity increases protein synthesis and decreases adipose tissue and pro-inflammatory factors (**Figure 2**).

## The Effect of Blood Flow Restriction (BFR) Exercises on Sarcopenic Obesity

Strength training with intensities greater than 60 to 70% of 1 repetition maximum is known as a golden standard intervention to maintain muscle function in aging. However, under clinical conditions, high-intensity exercise is not always possible for elderly and debilitated patients (155). Therefore, blood flow restriction (BFR) exercises are recommended, which are usually performed with a load of 15 to 30% of 1 repetition maximum, which in turn leads to an increase in muscle strength and hypertrophy as much as exercises with traditional heavy methods (156). Exercise studies have reported that resistance training with an intensity of more than 70% of 1 repetition maximum, is a strong stimulus for protein synthesis and hypertrophy. However, low-intensity resistance training with 20 to 50% of 1 repetition maximum, combined with BFR, results in similar increases in muscle size and strength compared to high-intensity resistance training (143).

One study found that BFR training combined with walking and traditional elastic band training in the elderly resulted in muscle hypertrophy of less than 1% to 2.6% per week and increased muscle strength of less than 1% to 5.9% per week. Finally, researchers stated that BFR training is a good intervention to reduce sarcopenia and increase muscle strength (156). Isometric exercise combined with BFR increases the concentrations of FGF-21, IL-6, IL-10, TNFα, and VEGF during exercise while decreasing oxygen saturation. These factors indicate the relationship of hypoxic environment affected by BFR with inflammatory cytokines. BFR training activates hypoxia-inducing factor (HIF-1α) by reducing oxygen delivery to the muscles and creating a hypoxic environment, which in turn increases HIF-1α by increasing NF-KB to the cell nucleus and then Increases the expression of inflammatory genes such as IL-6 and TNF-α. Neutrophils and macrophages in turn release IL-6 and TNFα, IGF-1, basal fibroblast growth factor (bFGF), transformer growth factor (TGF), and mechanical growth factor (MGF). Muscle hypertrophy is then stimulated by the IGF-1/AKT/mTORC1 pathway. On the other hand, ROS production *via* the IGF-1 and MAPK pathways stimulates the hypertrophic response to mTORC1 to regenerate muscle and activate satellite cells (157). In confirmation of this, exercise studies have shown that strength training with BFR improves skeletal muscle mass index, grip strength, C-reactive protein, oxidative stress, IL-6, and IGF-1 (155). However, after low-intensity BFR training, an increase in MAPK signaling (Akt ERK, p38 MAPK) is observed due to mechanical transmission or cellular stress to regulate skeletal muscle differentiation and proliferation. There is evidence that low-intensity BFR temporarily increases systemic GH and localized IGFs for proliferation and differentiation of satellite cells, both of which affect mTORC1 (158). In the elderly, skeletal muscle has not been shown to respond well to anabolic stimuli. For example, in response to acute resistance training, older people are less able to activate mTORC1 or increase muscle protein synthesis in the first 24 hours after exercise. However, BFR training in the elderly can overcome this dysfunction. BFR training stimulates muscle protein synthesis and mTORC1 signaling similar to high-intensity resistance training (143). A study reported that BFR training increases IGF-1, GH, and lactic acid in older people. This study suggested BFR exercises as an effective exercise method to improve the elderly and disabled (159). Electrical stimulation and BFR training also increase the expression of the FNDC5 protein, a source of irisin. The study found that BFR training is beneficial for the elderly and disabled (160). BFR exercises have been reported to increase irisin and decorin, which play an important role in muscle hypertrophy. These studies found BFR exercise useful for the elderly and obese (161, 162). In middle-aged women, in the traditional high-intensity resistance training group with low-intensity BFR resistance training compared to the control group, there is a significant increase in muscle volume, strength, and endurance. It was also reported that myostatin levels decrease after both types of resistance training (163). There is evidence also that in 68-year-old men, the concentrations of myostatin and follistatin in the BFR group decrease and increase (164). In aging people, Proteolytic genes such as FOXO3A, Atrogin-1, and MuRF-1 have been reported to decrease after 8 hours of BFR training (143). Therefore, based on the above studies, it can be said that resistance training with BFR increases myokines and muscle hypertrophy pathways, which in turn reduces protein degradation and muscle atrophy. There are limited studies on adipokines and BFR training. For example, an exercise study has shown that walking with BFR increases adiponectin in obese women (165). Another study showed that moderate-intensity endurance exercise in combination with BFR significantly increases the apelin receptor in aging mice (166). There is evidence that low-intensity endurance exercise plus BFR alters the expression of cardiac apelin receptors, leading to hypertrophy and ventricular conduction in older mice. It has also been shown that low-intensity exercise alone increases apelin receptor expression, but exercise with BFR increases apelin receptor expression more than exercise alone (167, 168). Therefore, further studies are needed to clarify the effect of resistance training with BFR on adipokines in people with sarcopenic obesity.

## Conclusion

Myokines and adipokines have been shown to undergo changes in people with sarcopenic obesity. For example, interleukin-6, as an adipokine, and myostatin increase with inactivity in people with sarcopenic obesity. These factors then cause muscle atrophy by decreasing the IGF-1/PI3k/Akt pathways, suppressing the mTOR pathway, and increasing the NF-KB and FOXO-1 pathways (6, 13, 45). In recent years, an exercise therapy approach has been proposed to combat sarcopenic obesity. Because after resistance and endurance training, IL-6 as a myokine, IGF-1, IL-10, and IL-15 increase, while MuRF and myostatin decrease with increasing PI3K/AKT, PGC1-α, and JNK pathways (43, 49, 117) (**Figure 1**). Then, the Ras/Raf/ERK pathway and the Akt/mTOR/p70S6K axis are activated by IGF-I, which can increase muscle cell proliferation, differentiation, and survival and prevent muscle atrophy (1). Then Akt inhibits FOXO to suppress protein degradation factors (13). Subsequently, with increasing IL-10, persistent inflammation in people with sarcopenic obesity decreases by suppressing macrophages and inflammatory factors such as TNF-α, IL-2, and IL-6 (13, 28). In addition, increasing IL-15 activates the JAK/STAT, PI3K/Akt pathways as well as the AMPK pathways (29). In general, exercise by activating these pathways reduces inflammation and muscle protein degradation and increases protein synthesis and hypertrophy. Other myokines that are reduced in people with sarcopenic obesity are irisin, FGF-21, and LIF. Decreasing these factors increases inflammation, mitochondrial dysfunction, increased white fat, decreased body temperature, and muscle atrophy. However, exercise with increased irisin *via* PGC1-α/TFAM/NRF1, IGF1-α/AKT/mTOR, and AMPK/PGC1-α/FNDC5 pathways lead to increased mitochondrogenesis, increased hypertrophy, and increased brown fat (51, 53) (**Figure 1**). In addition, increased FGF-21 and LIF during exercise may lead to decreased inflammation, increased mitochondrial function, increased thermogenesis, increased muscle hypertrophy, decreased atrophy, and the conversion of white fat to brown *via* the JAK/State/PI3K/Akt/mTOR (74, 80, 81).

On the other hand, an exercise-induced increase in leptin sensitivity through AMPK leads to a decrease in muscle triglycerides, myostatin suppression, and MuRF. Leptin then stimulates muscle hypertrophy through the IGF-1/AKT and JAK/STAT pathways (1, 83). In addition, exercise-induced adiponectin and apelin lead to mitochondrial biogenesis, decreased inflammation, decreased muscle atrophy, increased muscle hypertrophy, increased brown fat, decreased white fat, increased thermogenesis, decreased sarcopenic obesity, and increased myogenesis (82, 90, 101, 103). On the other hand, there are recommendations for using exercise therapy, especially BFR, to combat sarcopenia obesity. In this regard, it has been shown that BFR leads to an increase in IL-6, IL-10, irisin, IGF-1, adiponectin and apelin, and MAPK, MGF (143, 157, 160, 161, 165, 166). Then these factors reduce white fat, increase brown fat, increase hypertrophy, reduce atrophy, and increase thermogenesis in people with sarcopenic obesity (143, 157, 160, 161, 165, 166). Therefore, it seems that exercises such as aerobic exercise and resistance training with BFR can be a standard non-invasive intervention for people with sarcopenic obesity. While the effect of resistance exercise with BFR on adipokines due to lack of data needs further study. It is also recommended that future studies focus on the effects of exercise and BFR along with other methods such as gene therapy, dietary supplements, and cell therapy. In addition, studies can examine the synergistic effects of exercise-induced cellular mechanisms and BFR along with other invasive methods.

## Author Contributions

The author confirms being the sole contributor of this work and has approved it for publication.

## Conflict of Interest

The author declares that the research was conducted in the absence of any commercial or financial relationships that could be construed as a potential conflict of interest.

## Publisher’s Note

All claims expressed in this article are solely those of the authors and do not necessarily represent those of their affiliated organizations, or those of the publisher, the editors and the reviewers. Any product that may be evaluated in this article, or claim that may be made by its manufacturer, is not guaranteed or endorsed by the publisher.

## Abbreviations

mtDNA, mitochondrial DNA; ROS, reactive oxygen species; IL-6, interleukin-6; TNF-a, tumor necrosis factor a; CRP, C-reactive protein; insulin-like growth factor (IGF-1), FGF-21, fibroblast growth factor-21; ActRIIB , active type IIB receptor; MEF2, myocyte-specific enhancer factor; MyoD, myoblast-determining protein; p38MAPK mitogen-dependent pathogen p38; FOXO, Forkhead box O; MAFbx, muscle atrophy F-box; MURF-1, muscle RING finger 1; MUSA1, Muscle ubiquitin ligase of SCF complex in atrophy-1; SMART, specific for muscle atrophy and regulated by transcription; PMAT, perimuscular adipose tissue; PKB, Protein kinase B; JNK, c-Jun N-terminal kinases; PI3K, phosphatidylinositol 3-kinase; MGF, mechanical growth factor; FNDC5, fibronectin type-III domain containing protein 5; UCP1, uncoupling protein 1; LIF, Leukemia Inhibitory Factor; SCs, satellite cells; LPS, lipopolysaccharide; LepR, leptin receptor; JAK2, tyrosine kinase Janus kinase 2; BMI, body mass index; MMI, muscle mass index; adipoR1 and adipoR2,adiponectin 1 and 2 receptors; HIIT, high-intensity interval training; RET, Resistance exercise; BFR blood flow Restriction; HIF-1a, hypoxia-inducing factor; bFGF, basal fibroblast growth factor; TGF, transformer growth factor; MGF, mechanical growth factor; NF-KB, kappa B nuclear factor.

## References

[1] Priego T Martín A González-Hedström D Granado M López-Calderón A Cardalini D. Role of Hormones in Sarcopenia. Vitam Horm Elsevier (2021) 115:535–70. doi: 10.1016/bs.vh.2020.12.021 33706961

[2] Wannamethee SG Atkins JL. Muscle Loss and Obesity: The Health Implications of Sarcopenia and Sarcopenic Obesity. Proc Nutr Soc (2015) 74(4):405–12. doi: 10.1017/S002966511500169X 25913270

[3] China SP Pal S Chattopadhyay S Porwal K Kushwaha S Bhattacharyya S. Globular Adiponectin Reverses Osteo-Sarcopenia and Altered Body Composition in Ovariectomized Rats. Bone (2017) 105:75–86. doi: 10.1016/j.bone.2017.08.005 28811200

[4] Kirk B Feehan J Lombardi G Duque G. Muscle, Bone, and Fat Crosstalk: The Biological Role of Myokines, Osteokines, and Adipokines. Curr Osteoporosis Rep (2020) 18(4):388–400. doi: 10.1007/s11914-020-00599-y 32529456

[5] Cho J Choi Y Sajgalik P No M-H Lee S-H Kim S. Exercise as a Therapeutic Strategy for Sarcopenia in Heart Failure: Insights Into Underlying Mechanisms. Cells (2020) 9(10):2284. doi: 10.3390/cells9102284 PMC7602002 33066240

[6] Hong S-H Choi KM. Sarcopenic Obesity, Insulin Resistance, and Their Implications in Cardiovascular and Metabolic Consequences. Int J Mol Sci (2020) 21(2):494. doi: 10.3390/ijms21020494 PMC7013734 31941015

[7] Jensen GL. Inflammation: Roles in Aging and Sarcopenia. J Parenteral Enteral Nutr (2008) 32(6):656–9. doi: 10.1177/0148607108324585 18974248

[8] Liao C-D Tsauo J-Y Lin L-F Huang S-W Ku J-W Chou L-C. Effects of Elastic Resistance Exercise on Body Composition and Physical Capacity in Older Women With Sarcopenic Obesity: A CONSORT-Compliant Prospective Randomized Controlled Trial. Medicine (2017) 96(23):206–24. doi: 10.1097/MD.0000000000007115 PMC5466239 28591061

[9] Clark BC Clark LA Law TD. Resistance Exercise to Prevent and Manage Sarcopenia and Dynapenia. Annu Rev Gerontol Geriatrics (2016) 36(1):205–28. doi: 10.1200/jco.2017.75.7526 PMC4849483 27134329

[10] Dieli-Conwright CM Courneya KS Demark-Wahnefried W Sami N Lee K Buchanan TA. Effects of Aerobic and Resistance Exercise on Metabolic Syndrome, Sarcopenic Obesity, and Circulating Biomarkers in Overweight or Obese Survivors of Breast Cancer: A Randomized Controlled Trial. J Clin Oncol (2018) 36(9):875. doi: 10.1200/JCO.2017.75.7526 29356607 PMC5858524

[11] Bilski J Pierzchalski P Szczepanik M Bonior J Zoladz JA. Multifactorial Mechanism of Sarcopenia and Sarcopenic Obesity. Role of Physical Exercise, Microbiota and Myokines. Cells (2022) 11(1):160. doi: 10.3390/cells11010160 35011721 PMC8750433

[12] Scarpelli MC Bergamasco JGA de Barros Arruda EA Cook SB Libardi CA. Resistance Training With Partial Blood Flow Restriction in a 99-Year-Old Individual: A Case Report. Front Sports Active Living (2021) 3. doi: 10.3389/fspor.2021.671764 PMC8257955 34240050

[13] Guo A Li K Xiao Q. Sarcopenic Obesity: Myokines as Potential Diagnostic Biomarkers and Therapeutic Targets? Exp Gerontol (2020) 111022:1–60. doi: 10.1016/j.exger.2020.111022 32707318

[14] Foreman NA Hesse AS Ji LL. Redox Signaling and Sarcopenia: Searching for the Primary Suspect. Int J Mol Sci (2021) 22(16):9045. doi: 10.3390/ijms22169045 34445751 PMC8396474

[15] Rosa CGS Colares JR da Fonseca SRB dos Santos Martins G Miguel FM Dias AS. Sarcopenia, Oxidative Stress and Inflammatory Process in Muscle of Cirrhotic Rats–Action of Melatonin and Physical Exercise. Exp Mol Pathol (2021) 104662:1–9. doi: 10.1016/j.yexmp.2021.104662 34146550

[16] da Luz Scheffer D Latini A. Exercise-Induced Immune System Response: Anti-Inflammatory Status on Peripheral and Central Organs. Biochim Biophys Acta (BBA)-Mol Basis Dis (2020) 1866(10):165823. doi: 10.1016/j.bbadis.2020.165823 PMC7188661 32360589

[17] Payette H Roubenoff R Jacques PF Dinarello CA Wilson PW Abad LW. Insulin-Like Growth Factor-1 and Interleukin 6 Predict Sarcopenia in Very Old Community-Living Men and Women: The Framingham Heart Study. J Am Geriatrics Soc (2003) 51(9):1237–43. doi: 10.1046/j.1532-5415.2003.51407.x 12919235

[18] Nielsen AR Pedersen BK. The Biological Roles of Exercise-Induced Cytokines: IL-6, IL-8, and IL-15. Appl Physiol Nutr Metab (2007) 32(5):833–9. doi: 10.1139/H07-054 18059606

[19] Barbalho SM Flato UAP Tofano RJ Goulart R Guiguer EL Detregiachi CRP. Physical Exercise and Myokines: Relationships With Sarcopenia and Cardiovascular Complications. Int J Mol Sci (2020) 21(10):3607. doi: 10.3390/ijms21103607 PMC7279354 32443765

[20] Park J Bae J Lee J eds. Complex Exercise Improves Anti-Inflammatory and Anabolic Effects in Osteoarthritis-Induced Sarcopenia in Elderly Women. In: Healthcare. Naju: Multidisciplinary Digital Publishing Institute. 10.3390/healthcare9060711 PMC8230475 34200794

[21] Walston J Fedarko N Yang H Leng S Beamer B Espinoza S. The Physical and Biological Characterization of a Frail Mouse Model. J Gerontol Ser A: Biol Sci Med Sci (2008) 63(4):391–8. doi: 10.1093/gerona/63.4.391 PMC5903428 18426963

[22] Hacham M White RM Argov S Segal S Apte RN. Interleukin-6 and Interleukin-10 Are Expressed in Organs of Normal Young and Old Mice. Eur Cytokine Netw (2004) 15(1):37–46. 15217751

[23] Álvarez-Rodríguez L López-Hoyos M Muñoz-Cacho P Martínez-Taboada VM. Aging Is Associated With Circulating Cytokine Dysregulation. Cell Immunol (2012) 273(2):124–32. doi: 10.1016/j.cellimm.2012.01.001 22316526

[24] Rong Y-D Bian A-L Hu H-Y Ma Y Zhou X-Z. Study on Relationship Between Elderly Sarcopenia and Inflammatory Cytokine IL-6, Anti-Inflammatory Cytokine IL-10. BMC Geriatrics (2018) 18(1):1–6. doi: 10.1186/s12877-018-1007-9 30541467 PMC6292155

[25] Sakuma K Yamaguchi A. Sarcopenic Obesity and Endocrinal Adaptation With Age. Int J Endocrinol (2013) 2013:1–60. doi: 10.1155/2013/204164 PMC3639625 23690769

[26] Jenkins NT Padilla J Arce-Esquivel AA Bayless DS Martin JS Leidy HJ. Effects of Endurance Exercise Training, Metformin, and Their Combination on Adipose Tissue Leptin and IL-10 Secretion in OLETF Rats. J Appl Physiol (2012) 113(12):1873–83. doi: 10.1152/japplphysiol.00936.2012 PMC3544496 23019312

[27] Jung SH Park HS Kim K-S Choi WH Ahn CW Kim BT. Effect of Weight Loss on Some Serum Cytokines in Human Obesity: Increase in IL-10 After Weight Loss. J Nutr Biochem (2008) 19(6):371–5. doi: 10.1016/j.jnutbio.2007.05.007 17614271

[28] Batista M Rosa J Lopes R Lira F Martins E Yamashita A. Exercise Training Changes IL-10/TNF-α Ratio in the Skeletal Muscle of Post-MI Rats. Cytokine (2010) 49(1):102–8. doi: 10.1016/j.cyto.2009.10.007 19948415

[29] Aryana I Aprianta I Kuswardhani R. Role of Interleukin-15 in Sarcopenia: Future New Target Therapy. Int J Geriatr Gerontol: IJGG-104 DOI (2017) 10:1–8.

[30] Wang Q Hernández-Ochoa EO Viswanathan MC Blum ID Do DC Granger JM. CaMKII Oxidation is a Critical Performance/Disease Trade-Off Acquired at the Dawn of Vertebrate Evolution. Nat Commun (2021) 12(1):1–17. doi: 10.1038/s41467-021-23549-3 34039988 PMC8155201

[31] Aleksandrova K Koelman L Rodrigues CE. Dietary Patterns and Biomarkers of Oxidative Stress and Inflammation: A Systematic Review of Observational and Intervention Studies. Redox Biol (2021) 101869:1–16. doi: 10.1016/j.redox.2021.101869 PMC8113044 33541846

[32] Harper C Gopalan V Goh J. Exercise Rescues Mitochondrial Coupling in Aged Skeletal Muscle: A Comparison of Different Modalities in Preventing Sarcopenia. J Trans Med (2021) 19(1):1–17. doi: 10.1186/s12967-021-02737-1 PMC7885447 33593349

[33] Yalcin A Silay K Balik AR Avcioğlu G Aydin AS. The Relationship Between Plasma Interleukin-15 Levels and Sarcopenia in Outpatient Older People. Aging Clin Exp Res (2018) 30(7):783–90. doi: 10.1007/s40520-017-0848-y 29071664

[34] Riechman SE Balasekaran G Roth SM Ferrell RE. Association of Interleukin-15 Protein and Interleukin-15 Receptor Genetic Variation With Resistance Exercise Training Responses. J Appl Physiol (2004) 97(6):2214–9. doi: 10.1152/japplphysiol.00491.2004 15531573

[35] Ahima RS Park H-K. Connecting Myokines and Metabolism. Endocrinol Metab (2015) 30(3):235–45. doi: 10.3803/EnM.2015.30.3.235 PMC4595346 26248861

[36] Lutz CT Quinn LS. Sarcopenia, Obesity, and Natural Killer Cell Immune Senescence in Aging: Altered Cytokine Levels as a Common Mechanism. Aging (Albany NY) (2012) 4(8):535. doi: 10.18632/aging.100482 22935594 PMC3461341

[37] Krolopp JE Thornton SM Abbott MJ. IL-15 Activates the Jak3/STAT3 Signaling Pathway to Mediate Glucose Uptake in Skeletal Muscle Cells. Front Physiol (2016) 7:626. doi: 10.3389/fphys.2016.00626 28066259 PMC5167732

[38] Quinn LS Anderson BG Strait-Bodey L Stroud AM Argilés JM. Oversecretion of Interleukin-15 From Skeletal Muscle Reduces Adiposity. Am J Physiol-Endocrinol Metab (2009) 296(1):E191–202. doi: 10.1152/ajpendo.90506.2008 PMC2636988 19001550

[39] Baczek J Silkiewicz M Wojszel ZB. Myostatin as a Biomarker of Muscle Wasting and Other Pathologies-State of the Art and Knowledge Gaps. Nutrients (2020) 12(8):2401. doi: 10.3390/nu12082401 PMC7469036 32796600

[40] White TA LeBrasseur NK. Myostatin and Sarcopenia: Opportunities and Challenges-a Mini-Review. Gerontology (2014) 60(4):289–93. doi: 10.1159/000356740 24457615

[41] Léger B Derave W De Bock K Hespel P Russell AP. Human Sarcopenia Reveals an Increase in SOCS-3 and Myostatin and a Reduced Efficiency of Akt Phosphorylation. Rejuvenation Res (2008) 11(1):163–75B. doi: 10.1089/rej.2007.0588 18240972

[42] Consitt LA Clark B. The Vicious Cycle of Myostatin Signaling in Sarcopenic Obesity: Myostatin Role in Skeletal Muscle Growth, Insulin Signaling and Implications for Clinical Trials. J Frailty Aging (2018) 7(1):21–7. doi: 10.14283/jfa.2017.33 PMC6909929 29412438

[43] Bagheri R Moghadam BH Church DD Tinsley GM Eskandari M Moghadam BH. The Effects of Concurrent Training Order on Body Composition and Serum Concentrations of Follistatin, Myostatin and GDF11 in Sarcopenic Elderly Men. Exp Gerontol (2020) 133:110869. doi: 10.1016/j.exger.2020.110869 32035222

[44] Motahari Rad M Bijeh N Attarzadeh Hosseini SR Raouf Saeb A. The Effect of Two Concurrent Exercise Modalities on Serum Concentrations of FGF21, Irisin, Follistatin, and Myostatin in Men With Type 2 Diabetes Mellitus. Arch Physiol Biochem (2020), 1–10. 10.1080/13813455.2020.1829649 33044849

[45] Bataille S Chauveau P Fouque D Aparicio M Koppe L. Myostatin and Muscle Atrophy During Chronic Kidney Disease. Nephrol Dial Transplant (2020) 36:1986–93. doi: 10.1093/ndt/gfaa129 32974666

[46] Polyzos SA Margioris AN. Sarcopenic Obesity. Hormones (2018) 17(3):321–31. doi: 10.1007/s42000-018-0049-x 30014320

[47] Sakuma K Yamaguchi A. Sarcopenia and Cachexia: The Adaptations of Negative Regulators of Skeletal Muscle Mass. J Cachexia Sarcopenia Muscle (2012) 3(2):77–94. doi: 10.1007/s13539-011-0052-4 22476916 PMC3374017

[48] Chen HT Chung YC Chen YJ Ho SY Wu HJ. Effects of Different Types of Exercise on Body Composition, Muscle Strength, and IGF-1 in the Elderly With Sarcopenic Obesity. J Am Geriatrics Soc (2017) 65(4):827–32. doi: 10.1111/jgs.14722 28205203

[49] Annibalini G Lucertini F Agostini D Vallorani L Gioacchini A Barbieri E. Concurrent Aerobic and Resistance Training has Anti-Inflammatory Effects and Increases Both Plasma and Leukocyte Levels of IGF-1 in Late Middle-Aged Type 2 Diabetic Patients. Oxid Med Cell Longev (2017) 2017:1–11. doi: 10.1155/2017/3937842 PMC5497609 28713486

[50] Wang L-Z Guo Y-B Lou J-H. Effects of Home Exercise on Sarcopenia Obesity for Aging People. Chin J Rehabil Theory Pract (2019) 12:90–6.

[51] Choi HY Kim S Park JW Lee NS Hwang SY Huh JY. Implication of Circulating Irisin Levels With Brown Adipose Tissue and Sarcopenia in Humans. J Clin Endocrinol Metab (2014) 99(8):2778–85. doi: 10.1210/jc.2014-1195 24780049

[52] Colaianni G Mongelli T Colucci S Cinti S Grano M. Crosstalk Between Muscle and Bone via the Muscle-Myokine Irisin. Curr Osteoporosis Rep (2016) 14(4):132–7. doi: 10.1007/s11914-016-0313-4 27299471

[53] Paris MT Bell KE Mourtzakis M. Myokines and Adipokines in Sarcopenia: Understanding Cross-Talk Between Skeletal Muscle and Adipose Tissue and the Role of Exercise. Curr Opin Pharmacol (2020) 52:61–6. doi: 10.1016/j.coph.2020.06.003 32668398

[54] Zhao M Zhou X Yuan C Li R Ma Y Tang X. Association Between Serum Irisin Concentrations and Sarcopenia in Patients With Liver Cirrhosis: A Cross-Sectional Study. Sci Rep (2020) 10(1):1–9. doi: 10.1038/s41598-020-73176-z 32999391 PMC7527993

[55] Chang JS Kim TH Nguyen TT Park KS Kim N Kong ID. Circulating Irisin Levels as a Predictive Biomarker for Sarcopenia: A Cross-Sectional Community-Based Study. Geriatrics Gerontol Int (2017) 17(11):2266–73. doi: 10.1111/ggi.13030 28394089

[56] Lee MJ Lee SA Nam BY Park S Lee S-H Ryu HJ. Irisin, a Novel Myokine is an Independent Predictor for Sarcopenia and Carotid Atherosclerosis in Dialysis Patients. Atherosclerosis (2015) 242(2):476–82. doi: 10.1016/j.atherosclerosis.2015.08.002 26298738

[57] Oguz A Sahin M Tuzun D Kurutas EB Ulgen C Bozkus O. Irisin is a Predictor of Sarcopenic Obesity in Type 2 Diabetes Mellitus: A Cross-Sectional Study. Medicine (2021) 100(26):1–10. doi: 10.1097/MD.0000000000026529 PMC8257893 34190188

[58] Kim H-J So B Choi M Kang D Song W. Resistance Exercise Training Increases the Expression of Irisin Concomitant With Improvement of Muscle Function in Aging Mice and Humans. Exp Gerontol (2015) 70:11–7. doi: 10.1016/j.exger.2015.07.006 26183690

[59] Park H-S Kim HC Zhang D Yeom H Lim S-K. The Novel Myokine Irisin: Clinical Implications and Potential Role as a Biomarker for Sarcopenia in Postmenopausal Women. Endocrine (2019) 64(2):341–8. doi: 10.1007/s12020-018-1814-y 30570737

[60] Planella-Farrugia C Comas F Sabater-Masdeu M Moreno M Moreno-Navarrete JM Rovira O. Circulating Irisin and Myostatin as Markers of Muscle Strength and Physical Condition in Elderly Subjects. Front Physiol (2019) 10:871. doi: 10.3389/fphys.2019.00871 31354522 PMC6637304

[61] Romanello V. The Interplay Between Mitochondrial Morphology and Myomitokines in Aging Sarcopenia. Int J Mol Sci (2021) 22(1):91. doi: 10.3390/ijms22010091 PMC7796142 33374852

[62] Keipert S Ost M Johann K Imber F Jastroch M Van Schothorst EM. Skeletal Muscle Mitochondrial Uncoupling Drives Endocrine Cross-Talk Through the Induction of FGF21 as a Myokine. Am J Physiol-Endocrinol Metab (2014) 306(5):E469–E82. doi: 10.1152/ajpendo.00330.2013 24347058

[63] Oost LJ Kustermann M Armani A Blaauw B Romanello V. Fibroblast Growth Factor 21 Controls Mitophagy and Muscle Mass. J Cachexia Sarcopenia Muscle (2019) 10(3):630–42. doi: 10.1002/jcsm.12409 PMC6596457 30895728

[64] Jimenez V Jambrina C Casana E Sacristan V Muñoz S Darriba S. FGF21 Gene Therapy as Treatment for Obesity and Insulin Resistance. EMBO Mol Med (2018) 10(8):e8791. doi: 10.15252/emmm.201708791 29987000 PMC6079533

[65] Sanchis-Gomar F Pareja-Galeano H Mayero S Perez-Quilis C Lucia A. New Molecular Targets and Lifestyle Interventions to Delay Aging Sarcopenia. Front Aging Neurosci (2014) 6:156. doi: 10.3389/fnagi.2014.00156 25071565 PMC4078253

[66] Kim C-S Joe Y Choi H-S Back SH Park JW Chung HT. Deficiency of Fibroblast Growth Factor 21 Aggravates Obesity-Induced Atrophic Responses in Skeletal Muscle. J Inflamm (2019) 16(1):1–8. doi: 10.1186/s12950-019-0221-3 PMC6611052 31312114

[67] Bag Soytas R Suzan V Arman P Emiroglu Gedik T Unal D Cengiz M. Association of FGF-19 and FGF-21 Levels With Primary Sarcopenia. Geriatrics Gerontol Int (2021) 21(10):959–62. doi: 10.1111/ggi.14263 34405516

[68] Reinehr T Woelfle J Wunsch R Roth CL. Fibroblast Growth Factor 21 (FGF-21) and its Relation to Obesity, Metabolic Syndrome, and Nonalcoholic Fatty Liver in Children: A Longitudinal Analysis. J Clin Endocrinol Metab (2012) 97(6):2143–50. doi: 10.1210/jc.2012-1221 22438225

[69] Jung H-W Park JH Kim DA Jang I-Y Park SJ Lee JY. Association Between Serum FGF21 Level and Sarcopenia in Older Adults. Bone (2021) 145:115877. doi: 10.1016/j.bone.2021.115877 33571698

[70] Garneau L Aguer C. Role of Myokines in the Development of Skeletal Muscle Insulin Resistance and Related Metabolic Defects in Type 2 Diabetes. Diabetes Metab (2019) 45(6):505–16. doi: 10.1016/j.diabet.2019.02.006 30844447

[71] Khalafi M Alamdari KA Symonds ME Nobari H Carlos-Vivas J. Impact of Acute Exercise on Immediate and Following Early Post-Exercise FGF-21 Concentration in Adults: Systematic Review and Meta-Analysis. Hormones (2021) 20(1):23–33. doi: 10.1007/s42000-020-00245-3 33151509

[72] Kim H-J Song W. Resistance Training Increases Fibroblast Growth Factor-21 and Irisin Levels in the Skeletal Muscle of Zucker Diabetic Fatty Rats. J Exercise Nutr Biochem (2017) 21(3):50. doi: 10.20463/jenb.2017.0008 PMC5643202 29036766

[73] Oflazoglu U Caglar S Yılmaz HE Önal HT Varol U Salman T. The Relationship Between Sarcopenia Detected In Newly Diagnosed Colorectal Cancer Patients And FGF21, Irisin And CRP Levels. Res Sq [Preprint] (2021). Available at 10.21203/rs.3.rs-577872/v1 (Accessed February 14, 2022). 35277853

[74] Morville T Sahl RE Trammell SA Svenningsen JS Gillum MP Helge JW. Divergent Effects of Resistance and Endurance Exercise on Plasma Bile Acids, FGF19, and FGF21 in Humans. JCI Insight (2018) 3(15:1–12. doi: 10.1172/jci.insight.122737 PMC6129127 30089729

[75] Aryana IGPS Hapsari AAAR Kuswardhani RAT. Myokine Regulation as Marker of Sarcopenia in Elderly. Mol Cell Biomed Sci (2018) 2(2):38–47. doi: 10.21705/mcbs.v2i2.32

[76] Broholm C Brandt C Schultz NS Nielsen AR Pedersen BK Scheele C. Deficient Leukemia Inhibitory Factor Signaling in Muscle Precursor Cells From Patients With Type 2 Diabetes. Am J Physiol-Endocrinol Metab (2012) 303(2):E283–E92. doi: 10.1152/ajpendo.00586.2011 22649064

[77] Pratesi A Tarantini F Di Bari M. Skeletal Muscle: An Endocrine Organ. Clin cases Mineral Bone Metab (2013) 10(1):11. doi: 10.11138/ccmbm/2013.10.1.011 PMC3710002 23858303

[78] Huh JY. The Role of Exercise-Induced Myokines in Regulating Metabolism. Arch Pharmacal Res (2018) 41(1):14–29. doi: 10.1007/s12272-017-0994-y 29177585

[79] Catalán V Frühbeck G Gómez-Ambrosi J. Inflammatory and Oxidative Stress Markers in Skeletal Muscle of Obese Subjects. Obesity: Elsevier (2018) p:163–89. doi: 10.1016/b978-0-12-812504-5.00008-8

[80] Leuchtmann AB Adak V Dilbaz S Handschin C. The Role of the Skeletal Muscle Secretome in Mediating Endurance and Resistance Training Adaptations. Front Physiol (2021) 1296. doi: 10.3389/fphys.2021.709807 PMC8387622 34456749

[81] Broholm C Laye MJ Brandt C Vadalasetty R Pilegaard H Pedersen BK. LIF is a Contraction-Induced Myokine Stimulating Human Myocyte Proliferation. J Appl Physiol (2011) 111(1):251–9. doi: 10.1152/japplphysiol.01399.2010 21527666

[82] Kalinkovich A Livshits G. Sarcopenic Obesity or Obese Sarcopenia: A Cross Talk Between Age-Associated Adipose Tissue and Skeletal Muscle Inflammation as a Main Mechanism of the Pathogenesis. Ageing Res Rev (2017) 35:200–21. doi: 10.1016/j.arr.2016.09.008 27702700

[83] Peng J Yin L Wang X. Central and Peripheral Leptin Resistance in Obesity and Improvements of Exercise. Hormones Behav (2021) 133:105006. doi: 10.1016/j.yhbeh.2021.105006 34087669

[84] Hamrick MW. Role of the Cytokine-Like Hormone Leptin in Muscle-Bone Crosstalk With Aging. J Bone Metab (2017) 24(1):1–8. doi: 10.11005/jbm.2017.24.1.1 28326295 PMC5357607

[85] Nilsson MI Dobson JP Greene NP Wiggs MP Shimkus KL Wudeck EV. Abnormal Protein Turnover and Anabolic Resistance to Exercise in Sarcopenic Obesity. FASEB J (2013) 27(10):3905–16. doi: 10.1096/fj.12-224006 23804240

[86] Manoy P Anomasiri W Yuktanandana P Tanavalee A Ngarmukos S Tanpowpong T. Elevated Serum Leptin Levels Are Associated With Low Vitamin D, Sarcopenic Obesity, Poor Muscle Strength, and Physical Performance in Knee Osteoarthritis. Biomarkers (2017) 22(8):723–30. doi: 10.1080/1354750X.2017.1315615 28374624

[87] Kim H Kim M Kojima N Fujino K Hosoi E Kobayashi H. Exercise and Nutritional Supplementation on Community-Dwelling Elderly Japanese Women With Sarcopenic Obesity: A Randomized Controlled Trial. J Am Med Dir Assoc (2016) 17(11):1011–9. doi: 10.1016/j.jamda.2016.06.016 27544583

[88] Park AJ Battaglino RA Nguyen NM Morse LR. Associations Between Lean Mass and Leptin in Men With Chronic Spinal Cord Injury: Results From the FRASCI-Muscle Study. PloS One (2018) 13(6):e0198969. doi: 10.1371/journal.pone.0198969 29949600 PMC6021064

[89] Kang S Kim KB Shin KO. Exercise Training Improve Leptin Sensitivity in Peripheral Tissue of Obese Rats. Biochem Biophys Res Commun (2013) 435(3):454–9. doi: 10.1016/j.bbrc.2013.05.007 23669042

[90] Abou-Samra M Selvais CM Dubuisson N Brichard SM. Adiponectin and its Mimics on Skeletal Muscle: Insulin Sensitizers, Fat Burners, Exercise Mimickers, Muscling Pills… or Everything Together? Int J Mol Sci (2020) 21(7):2620. doi: 10.3390/ijms21072620 PMC7178193 32283840

[91] Inoue A Cheng XW Huang Z Hu L Kikuchi R Jiang H. Exercise Restores Muscle Stem Cell Mobilization, Regenerative Capacity and Muscle Metabolic Alterations via Adiponectin/AdipoR1 Activation in SAMP10 Mice. J Cachexia Sarcopenia Muscle (2017) 8(3):370–85. doi: 10.1002/jcsm.12166 PMC5476856 27897419

[92] O’Leary MF Wallace GR Davis ET Murphy DP Nicholson T Bennett AJ. Obese Subcutaneous Adipose Tissue Impairs Human Myogenesis, Particularly in Old Skeletal Muscle, via Resistin-Mediated Activation of Nfκb. Sci Rep (2018) 8(1):1–13. doi: 10.1038/s41598-018-33840-x 30337633 PMC6193975

[93] Biolo G Di Girolamo FG Breglia A Chiuc M Baglio V Vinci P. Inverse Relationship Between “A Body Shape Index”(ABSI) and Fat-Free Mass in Women and Men: Insights Into Mechanisms of Sarcopenic Obesity. Clin Nutr (2015) 34(2):323–7. doi: 10.1016/j.clnu.2014.03.015 24814384

[94] Botero JP Shiguemoto GE Prestes J Marin CT Do Prado W Pontes C. Effects of Long-Term Periodized Resistance Training on Body Composition, Leptin, Resistin and Muscle Strength in Elderly Post-Menopausal Women. J Sports Med Phys Fitness (2013) 53(3):289–94. 23715254

[95] Düzova H Güllü E Çiçek G Köksal B Kayhan B Güllü A. The Effect of Exercise Induced Weight Loss on Myokines and Adipokines in Overweight Sedentary Females Steps Aerobics vs Jogging Walking Exercises. J Sports Med Phys Fitnes (2016), 2–27. 10.23736/S0022-4707.16.06565-8 27901337

[96] Jamurtas AZ Stavropoulos-Kalinoglou A Koutsias S Koutedakis Y Fatouros I. Adiponectin, Resistin, and Visfatin in Childhood Obesity and Exercise. Pediatr Exercise Sci (2015) 27(4):454–62. doi: 10.1123/pes.2014-0072 25902558

[97] Azuma K Katsukawa F Oguchi S Murata M Yamazaki H Shimada A. Correlation Between Serum Resistin Level and Adiposity in Obese Individuals. Obes Res (2003) 11(8):997–1001. doi: 10.1038/oby.2003.137 12917505

[98] Aghapour A Farzanegi P. Effect of Six-Week Aerobic Exercise on Chemerin and Resistin Concentration in Hypertensive Postmenopausal Women. Electron Physician (2013) 5(1):623. doi: 10.14661/2013.623-630 26120393 PMC4477779

[99] Afshounpour MT Habibi A Ranjbar R. Impact of Combined Exercise Training on Plasma Concentration of Apelin, Resistin and Insulin Resistance in Patients With Type 2 Diabetics’ Male. Hormozgan Med J (2016) 20(3):158–69.

[100] Son JS Kim HJ Son Y Lee H Chae SA Seong JK. Effects of Exercise-Induced Apelin Levels on Skeletal Muscle and Their Capillarization in Type 2 Diabetic Rats. Muscle Nerve (2017) 56(6):1155–63. doi: 10.1002/mus.25596 28164323

[101] Vinel C Lukjanenko L Batut A Deleruyelle S Pradere J-P Le Gonidec S. The Exerkine Apelin Reverses Age-Associated Sarcopenia. Nat Med (2018) 24(9):1360–71. doi: 10.1038/s41591-018-0131-6 30061698

[102] Takada S Sabe H Kinugawa S. Abnormalities of Skeletal Muscle, Adipocyte Tissue, and Lipid Metabolism in Heart Failure: Practical Therapeutic Targets. Front Cardiovasc Med (2020) 7:79. doi: 10.3389/fcvm.2020.00079 32478098 PMC7235191

[103] Kwon JH Moon KM Min K-W eds. Exercise-Induced Myokines can Explain the Importance of Physical Activity in the Elderly: An Overview. In: Healthcare. Ulsan: Multidisciplinary Digital Publishing Institute. 10.3390/healthcare8040378 PMC7712334 33019579

[104] Chen Y-Y Chiu Y-L Kao T-W Peng T-C Yang H-F Chen W-L. Cross-Sectional Associations Among P3NP, HtrA, Hsp70, Apelin and Sarcopenia in Taiwanese Population. BMC Geriatrics (2021) 21(1):1–9. doi: 10.1186/s12877-021-02146-5 33743591 PMC7980650

[105] Chen TC Huang T-H Tseng W-C Tseng K-W Hsieh C-C Chen M-Y. Changes in Plasma C1q, Apelin and Adropin Concentrations in Older Adults After Descending and Ascending Stair Walking Intervention. Sci Rep (2021) 11(1):1–11. doi: 10.1038/s41598-021-96631-x 34480035 PMC8417101

[106] Son JS Zhao L Chen Y Chen K Chae SA de Avila JM. Maternal Exercise via Exerkine Apelin Enhances Brown Adipogenesis and Prevents Metabolic Dysfunction in Offspring Mice. Sci Adv (2020) 6(16):eaaz0359. doi: 10.1126/sciadv.aaz0359 32494609 PMC7164955

[107] Cornish SM Bugera EM Duhamel TA Peeler JD Anderson JE. A Focused Review of Myokines as a Potential Contributor to Muscle Hypertrophy From Resistance-Based Exercise. Eur J Appl Physiol (2020) 120(5):941–59. doi: 10.1007/s00421-020-04337-1 32144492

[108] Fukada S-I Nakamura A. Exercise/Resistance Training and Muscle Stem Cells. Endocrinol Metab (2021) 36(4):737. doi: 10.3803/EnM.2021.401 PMC8419599 34372625

[109] Brocca L Toniolo L Reggiani C Bottinelli R Sandri M Pellegrino MA. FoxO-Dependent Atrogenes Vary Among Catabolic Conditions and Play a Key Role in Muscle Atrophy Induced by Hindlimb Suspension. J Physiol (2017) 595(4):1143–58. doi: 10.1113/JP273097 PMC5309360 27767211

[110] Sandri M Sandri C Gilbert A Skurk C Calabria E Picard A. Foxo Transcription Factors Induce the Atrophy-Related Ubiquitin Ligase Atrogin-1 and Cause Skeletal Muscle Atrophy. Cell (2004) 117(3):399–412. doi: 10.1016/S0092-8674(04)00400-3 15109499 PMC3619734

[111] Lee K Ochi E Song H Nakazato K. Activation of AMP-Activated Protein Kinase Induce Expression of FoxO1, FoxO3a, and Myostatin After Exercise-Induced Muscle Damage. Biochem Biophys Res Commun (2015) 466(3):289–94. doi: 10.1016/j.bbrc.2015.08.126 26342801

[112] Liang J Zhang H Zeng Z Wu L Zhang Y Guo Y. Lifelong Aerobic Exercise Alleviates Sarcopenia by Activating Autophagy and Inhibiting Protein Degradation via the AMPK/PGC-1α Signaling Pathway. Metabolites (2021) 11(5):323. doi: 10.3390/metabo11050323 34069829 PMC8157243

[113] Romanello V Sandri M. Mitochondria Quality Control and Muscle Mass Maintenance. Front Physiol (2016) 6:422. doi: 10.3389/fphys.2015.00422 26793123 PMC4709858

[114] Wang Q Hu J Liu Y Li J Liu B Li M. Aerobic Exercise Improves Synaptic-Related Proteins of Diabetic Rats by Inhibiting FOXO1/NF-κb/NLRP3 Inflammatory Signaling Pathway and Ameliorating PI3K/Akt Insulin Signaling Pathway. J Mol Neurosci (2019) 69(1):28–38. doi: 10.1007/s12031-019-01302-2 31111330

[115] Allen DL Unterman TG. Regulation of Myostatin Expression and Myoblast Differentiation by FoxO and SMAD Transcription Factors. Am J Physiol-Cell Physiol (2007) 292(1):C188–C99. doi: 10.1152/ajpcell.00542.2005 16885393

[116] Wu L Wang Q Guo F Zhou Y Ji H Liu F. Activation of FoxO1/PGC-1α Prevents Mitochondrial Dysfunction and Ameliorates Mesangial Cell Injury in Diabetic Rats. Mol Cell Endocrinol (2015) 413:1–12. doi: 10.1016/j.mce.2015.06.007 26123583

[117] Meng S-J Yu L-J. Oxidative Stress, Molecular Inflammation and Sarcopenia. Int J Mol Sci (2010) 11(4):1509–26. doi: 10.3390/ijms11041509 PMC2871128 20480032

[118] Zhu S Tian Z Torigoe D Zhao J Xie P Sugizaki T. Aging-And Obesity-Related Peri-Muscular Adipose Tissue Accelerates Muscle Atrophy. PloS One (2019) 14(8):e0221366. doi: 10.1371/journal.pone.0221366 31442231 PMC6707561

[119] Ropelle ER Pauli JR Cintra DE Frederico MJ De Pinho RA Velloso LA. Acute Exercise Modulates the Foxo1/PGC-1α Pathway in the Liver of Diet-Induced Obesity Rats. J Physiol (2009) 587(9):2069–76. doi: 10.1113/jphysiol.2008.164202 PMC2689344 19273580

[120] Bowen TS Schuler G Adams V. Skeletal Muscle Wasting in Cachexia and Sarcopenia: Molecular Pathophysiology and Impact of Exercise Training. J Cachexia Sarcopenia Muscle (2015) 6(3):197–207. doi: 10.1002/jcsm.12043 26401465 PMC4575550

[121] Liu P-J Hu Y-S Wang M-J Kang L. Nutrient Weight Against Sarcopenia: Regulation of the IGF-1/PI3K/Akt/FOXO Pathway in Quinoa Metabolites. Curr Opin Pharmacol (2021) 61:136–41. doi: 10.1016/j.coph.2021.10.001 34801804

[122] Seo DY Hwang BG. Effects of Exercise Training on the Biochemical Pathways Associated With Sarcopenia. Phys Activity Nutr (2020) 24(3):32. doi: 10.20463/pan.2020.0019 PMC7669465 33108716

[123] Stefanetti RJ Lamon S Rahbek SK Farup J Zacharewicz E Wallace MA. Influence of Divergent Exercise Contraction Mode and Whey Protein Supplementation on Atrogin-1, MuRF1, and FOXO1/3A in Human Skeletal Muscle. J Appl Physiol (2014) 116(11):1491–502. doi: 10.1152/japplphysiol.00136.2013 24458747

[124] Bedada FB Ntekim OE Nwulia EO Fungwe TV Nadarajah SR Obisesan TO. Exercise Training-Increased FBXO32 and FOXO1 in a Gender-Dependent Manner in Mild Cognitively Impaired African Americans: GEMS-1 Study. Front Aging Neurosci (2021) 13:174. doi: 10.3389/fnagi.2021.641758 PMC8079639 33935685

[125] Tang L Cao W Zhao T Yu K Sun L Guo J. Weight-Bearing Exercise Prevents Skeletal Muscle Atrophy in Ovariectomized Rats. J Physiol Biochem (2021) 77(2):273–81. doi: 10.1007/s13105-021-00794-0 33788149

[126] Li T-C Wu C-W Li C-I Wu F-Y Liao L-N Liu C-S. Interactions Among IGF-1, AKT2, FOXO1, and FOXO3 Variations and Between Genes and Physical Activities on Physical Performance in Community-Dwelling Elders. PloS One (2020) 15(9):e0239530. doi: 10.1371/journal.pone.0239530 32986769 PMC7521683

[127] Lessard SJ MacDonald TL Pathak P Han MS Coffey VG Edge J. JNK Regulates Muscle Remodeling via Myostatin/SMAD Inhibition. Nat Commun (2018) 9(1):1–14. doi: 10.1038/s41467-018-05439-3 30072727 PMC6072737

[128] Yang J Sun L Fan X Yin B Kang Y Tang L. Effect of Exercise on Bone in Poorly Controlled Type 1 Diabetes Mediated by the ActRIIB/Smad Signaling Pathway. Exp Ther Med (2018) 16(4):3686–93. doi: 10.3892/etm.2018.6601 PMC6143879 30233727

[129] Mehdipoor M Damirchi A Tousi SMTR Babaei P. Concurrent Vitamin D Supplementation and Exercise Training Improve Cardiac Fibrosis via TGF-β/Smad Signaling in Myocardial Infarction Model of Rats. J Physiol Biochem (2021) 77(1):75–84. doi: 10.1007/s13105-020-00778-6 33428175

[130] Wang S-Q Li D Yuan Y. Long-Term Moderate Intensity Exercise Alleviates Myocardial Fibrosis in Type 2 Diabetic Rats via Inhibitions of Oxidative Stress and TGF-β1/Smad Pathway. J Physiol Sci (2019) 69(6):861–73. doi: 10.1007/s12576-019-00696-3 PMC10716963 31392590

[131] Sartori R Romanello V Sandri M. Mechanisms of Muscle Atrophy and Hypertrophy: Implications in Health and Disease. Nat Commun (2021) 12(1):1–12. doi: 10.1038/s41467-020-20123-1 33436614 PMC7803748

[132] Bar-Shai M Carmeli E Reznick AZ. The Role of NF-κb in Protein Breakdown in Immobilization, Aging, and Exercise: From Basic Processes to Promotion of Health. Ann New York Acad Sci (2005) 1057(1):431–47. doi: 10.1196/annals.1356.034 16399911

[133] Liu H-W Chang S-J. Moderate Exercise Suppresses NF-κb Signaling and Activates the SIRT1-AMPK-Pgc1α Axis to Attenuate Muscle Loss in Diabetic Db/Db Mice. Front Physiol (2018) 9:636. doi: 10.3389/fphys.2018.00636 29896118 PMC5987703

[134] Gielen S Adams V Möbius-Winkler S Linke A Erbs S Yu J. Anti-Inflammatory Effects of Exercise Training in the Skeletal Muscle of Patients With Chronic Heart Failure. J Am Coll Cardiol (2003) 42(5):861–8. doi: 10.1016/S0735-1097(03)00848-9 12957433

[135] Khor SC Abdul Karim N Wan Ngah WZ Mohd Yusof YA Makpol S. Vitamin E in Sarcopenia: Current Evidences on its Role in Prevention and Treatment. Oxid Med Cell Longevity (2014) 2014:1–17. doi: 10.1155/2014/914853 PMC4109111 25097722

[136] Shenas NY Peeri M Delfan M. The Effect of 10 Weeks Endurance Training on Protein Levels of NF-kB and Gene Expression of Atrogin-1 and MuRF-1 in Cardiac Myocytes of Female. Med J Tabriz Univ Med Sci (2021) 43(1):134–41. doi: 10.34172/mj.2021.038

[137] Yazdanshenas N Peeri M Delfan M. Effect of 10 Weeks of High-Intensity Interval Training on Protein Levels of NF-kB and Expression of Atrogin-1 and MuRF-1 in Cardiomyocytes of Female Mice With Breast Cancer. Iran J Breast Dis (2020) 13(3):63–71. doi: 10.30699/ijbd.13.3.62

[138] Kawada S Ishii N. Skeletal Muscle Hypertrophy After Chronic Restriction of Venous Blood Flow in Rats. Med Sci Sports Exercise (2005) 37(7):1144. doi: 10.1249/01.mss.0000170097.59514.bb 16015131

[139] Luo L Lu A-M Wang Y Hong A Chen Y Hu J. Chronic Resistance Training Activates Autophagy and Reduces Apoptosis of Muscle Cells by Modulating IGF-1 and its Receptors, Akt/mTOR and Akt/FOXO3a Signaling in Aged Rats. Exp Gerontol (2013) 48(4):427–36. doi: 10.1016/j.exger.2013.02.009 23419688

[140] Barberi L Scicchitano BM Musaro A. Molecular and Cellular Mechanisms of Muscle Aging and Sarcopenia and Effects of Electrical Stimulation in Seniors. Eur J Trans Myology (2015) 25(4):231–7. doi: 10.4081/ejtm.2015.5227 PMC4748976 26913161

[141] Adamo ML Farrar RP. Resistance Training, and IGF Involvement in the Maintenance of Muscle Mass During the Aging Process. Ageing Res Rev (2006) 5(3):310–31. doi: 10.1016/j.arr.2006.05.001 16949353

[142] Lee S Barton ER Sweeney HL Farrar RP. Viral Expression of Insulin-Like Growth Factor-I Enhances Muscle Hypertrophy in Resistance-Trained Rats. J Appl Physiol (2004) 96(3):1097–104. doi: 10.1152/japplphysiol.00479.2003 14766764

[143] Walker DK Dickinson JM Timmerman KL Drummond MJ Reidy PT Fry CS. Exercise, Amino Acids and Aging in the Control of Human Muscle Protein Synthesis. Med Sci Sports Exercise (2011) 43(12):2249. doi: 10.1249/MSS.0b013e318223b037 PMC3289515 21606874

[144] Gomes MJ Martinez PF Pagan LU Damatto RL Cezar MDM Lima ARR. Skeletal Muscle Aging: Influence of Oxidative Stress and Physical Exercise. Oncotarget (2017) 8(12):20428. doi: 10.18632/oncotarget.14670 28099900 PMC5386774

[145] Konopka AR Douglass MD Kaminsky LA Jemiolo B Trappe TA Trappe S. Molecular Adaptations to Aerobic Exercise Training in Skeletal Muscle of Older Women. J Gerontol Ser: Biomed Sci Med Sci (2010) 65(11):1201–7. doi: 10.1093/gerona/glq109 PMC2954235 20566734

[146] Nygaard H Slettaløkken G Vegge G Hollan I Whist JE Strand T. Irisin in Blood Increases Transiently After Single Sessions of Intense Endurance Exercise and Heavy Strength Training. PloS One (2015) 10(3):e0121367. doi: 10.1371/journal.pone.0121367 25781950 PMC4363689

[147] Picca A Calvani R Bossola M Allocca E Menghi A Pesce V. Update on Mitochondria and Muscle Aging: All Wrong Roads Lead to Sarcopenia. Biol Chem (2018) 399(5):421–36. doi: 10.1515/hsz-2017-0331 29384724

[148] Navas-Enamorado I Bernier M Brea-Calvo G de Cabo R. Influence of Anaerobic and Aerobic Exercise on Age-Related Pathways in Skeletal Muscle. Ageing Res Rev (2017) 37:39. doi: 10.1016/j.arr.2017.04.005 28487241 PMC5549001

[149] Jia D Cai M Xi Y Du S. Interval Exercise Training Increases LIF Expression and Prevents Myocardial Infarction-Induced Skeletal Muscle Atrophy in Rats. Life Sci (2018) 193:77–86. doi: 10.1016/j.lfs.2017.12.009 29223542

[150] Scisciola L Fontanella RA Cataldo V Paolisso G Barbieri M. Sarcopenia and Cognitive Function: Role of Myokines in Muscle Brain Cross-Talk. Life (2021) 11(2):173. doi: 10.3390/life11020173 33672427 PMC7926334

[151] Fatouros I Tournis S Leontsini D Jamurtas A Sxina M Thomakos P. Leptin and Adiponectin Responses in Overweight Inactive Elderly Following Resistance Training and Detraining are Intensity Related. J Clin Endocrinol Metab (2005) 90(11):5970–7. doi: 10.1210/jc.2005-0261 16091494

[152] Prestes J da Cunha Nascimento D de Sousa Neto IV Tibana RA Shiguemoto GE de Andrade Perez SE. The Effects of Muscle Strength Responsiveness to Periodized Resistance Training on Resistin, Leptin, and Cytokine in Elderly Postmenopausal Women. J Strength Conditioning Res (2018) 32(1):113–20. doi: 10.1519/JSC.0000000000001718 28661971

[153] Prestes J Shiguemoto G Botero JP Frollini A Dias R Leite R. Effects of Resistance Training on Resistin, Leptin, Cytokines, and Muscle Force in Elderly Post-Menopausal Women. J Sports Sci (2009) 27(14):1607–15. doi: 10.1080/02640410903352923 19967592

[154] Mijwel S. The Effect of Resistance Training on Molecular Mechanisms Responsible for Muscle Protein Breakdown in Healthy Old Men. School Health Med Sci (2012), 1–31.

[155] Lopes KG Bottino DA Farinatti P de Souza M Maranhão PA de Araujo CMS. Strength Training With Blood Flow Restriction–a Novel Therapeutic Approach for Older Adults With Sarcopenia? A Case Rep Clin Interventions Aging (2019) 14:1461. doi: 10.2147/CIA.S206522 PMC6698614 31616137

[156] Hackney KJ Brown L Stone KA Tennent DJ. The Role of Blood Flow Restriction Training to Mitigate Sarcopenia, Dynapenia, and Enhance Clinical Recovery. Techniques Orthopaedics (2018) 33(2):98–105. doi: 10.1097/BTO.0000000000000271

[157] Rossi FE de Freitas MC Zanchi NE Lira FS Cholewa JM. The Role of Inflammation and Immune Cells in Blood Flow Restriction Training Adaptation: A. Myokines, Adipokines, Cytokines in Muscle Pathophysiology. Front Physiol (2020) 9:1–9. doi: 10.3389/fphys.2018.01376 PMC6189414 30356748

[158] Lambert BS Hedt C Moreno M Harris JD McCulloch P. Blood Flow Restriction Therapy for Stimulating Skeletal Muscle Growth: Practical Considerations for Maximizing Recovery in Clinical Rehabilitation Settings. Techniques Orthopaedics (2018) 33(2):89–97. doi: 10.1097/BTO.0000000000000275

[159] Ortiz A Arguello E. Blood Flow Restriction as an Exercise Alternative to Ameliorate the Effects of Aging. Curr Geriatr Rep (2020) 9:1–6. doi: 10.1007/s13670-020-00323-9

[160] Nakajima T Koide S Yasuda T Hasegawa T Yamasoba T Obi S. Muscle Hypertrophy Following Blood Flow-Restricted, Low-Force Isometric Electrical Stimulation in Rat Tibialis Anterior: Role for Muscle Hypoxia. J Appl Physiol (2018) 125(1):134–45. doi: 10.1152/japplphysiol.00972.2017 29565774

[161] Helzer D. Acute Responses to Exercise With Blood Flow Restriction: A Systematic Review. A Projet Presented to the Faculty of California State Polytechnic University, Pomona (2021). pp. 1–206.

[162] Silva JCG Pereira Neto EA Pfeiffer PAS Neto GR Rodrigues AS Bemben MG. Acute and Chronic Responses of Aerobic Exercise With Blood Flow Restriction: A Systematic Review. Front Physiol (2019) 10:1239. doi: 10.3389/fphys.2019.01239 31636569 PMC6787286

[163] Mohammadi Gonbad G Farzaneh Hesari A Abbaszadeh Sourati H. Comparison of the Effects of Resistance Training With Blood Flow Restriction and Traditional Resistance Training on Myostatin, Muscle Mass and Some Physiological Factors in Middle-Aged Women: A Clinical Trial. J Rafsanjan Univ Med Sci (2019) 18(1):31–42.

[164] Shikhi Pir Kohi Z Zakeri P Dehkhoda M Mirakhori Z Amani-Shalamzari S. The Effect of Six Weeks of Functional Training With Blood Flow Restriction on Myostatin to Folistatin Ratio and Physical Fitness in Elderly Men. J Appl Exercise Physiol (2019) 15(30):227–43. doi: 10.22080/jaep.2019.17016.1901

[165] Oh D-H Kim J-H Zhang S-A Lee J-K. Effect of 4 Weeks' Walking Exercise With Blood Flow Restriction on Insulin Resistance, Adipokines and Gut Hormones in Middle Aged Obese Women. J Korea Academia-Industrial Cooperation Soc (2018) 19(3):489–98. doi: 10.5762/kais.2018.19.3.489

[166] Raji-Amirhasani A Joukar S Naderi-Boldaji V Bejeshk M-A. Mild Exercise Along With Limb Blood-Flow Restriction Modulates the Electrocardiogram, Angiotensin, and Apelin Receptors of the Heart in Aging Rats. Iranian J Basic Med Sci (2018) 21(6):558. doi: 10.22038/ijbms.2018.24796.6165 PMC6015241 29942444

[167] Bejeshk M-A Joukar S Shahouzehi B Asadi-shekari M Rajizadeh M Raji-amirhasani A. Combinatorial Effect of Lower Extremity Blood Flow Restriction and Low Intensity Endurance Exercise on Aorta of Old Male Rats: Histomorphological and Molecular Approach. Artery Res (2018) 24:22–31. doi: 10.1016/j.artres.2018.10.226

[168] Naderi-Boldaji V Joukar S Noorafshan A Raji-Amirhasani A Naderi-Boldaji S Bejeshk M-A. The Effect of Blood Flow Restriction Along With Low-Intensity Exercise on Cardiac Structure and Function in Aging Rat: Role of Angiogenesis. Life Sci (2018) 209:202–9. doi: 10.1016/j.lfs.2018.08.015 30096385

